# HOXD9 promotes the growth, invasion and metastasis of gastric cancer cells by transcriptional activation of RUFY3

**DOI:** 10.1186/s13046-019-1399-1

**Published:** 2019-09-23

**Authors:** Huiqiong Zhu, Weiyu Dai, Jiaying Li, Li Xiang, Xiaosheng Wu, Weimei Tang, Yaying Chen, Qiong Yang, Mengwei Liu, Yizhi Xiao, Wenjing Zhang, Jianjiao Lin, Jing Wang, Guangnan Liu, Yong Sun, Ping Jiang, Guoxin Li, Aimin Li, Side Liu, Ye Chen, Jide Wang

**Affiliations:** 1 Guangdong Provincial Key Laboratory of Gastroenterology, Department of Gastroenterology, Nanfang Hospital, Southern Medical University, Guangzhou, 510515 China; 2 Department of Gastroenterology, Longgang District People’s Hospital, Shenzhen, 518172 China; 3 Department of Gastroenterology, The Third Affiliated Hospital of Guangzhou Medical University, Guangzhou, 510515 China; 4 Department of Medical Oncology, the First people’s Hospital of Yunnan Province, Medical School of Kunming University of Science and Technology, Kunming, 650032 China; 5 Department of General Surgery, Nanfang Hospital, Southern Medical University, Guangzhou, 510515 China; 6 Department of Digestive Medicine, Nanfang Hospital, Southern Medical University, Guangzhou, 510515 Guangdong Province China

**Keywords:** HOXD9, RUFY3, Gastric cancer, Proliferation, Metastasis

## Abstract

**Background:**

The transcription factor HOXD9 is one of the members of the HOX family, which plays an important role in neoplastic processes. However, the role of HOXD9 in the growth and metastasis of gastric cancer (GC) remains to be elucidated.

**Methods:**

In vitro functional role of HOXD9 and RURY3 in GC cells was determined using the TMA-based immunohistochemistry, western blot, EdU incorporation, gelatin zymography, luciferase, chromatin Immunoprecipitation (ChIP) and cell invasion assays. In vivo tumor growth and metastasis were conducted in nude mice.

**Results:**

HOXD9 is overexpressed in GC cells and tissues***.*** The high expression of HOXD9 was correlated with poor survival in GC patients. Functionally, HOXD9 expression significantly promoted the proliferation, invasion and migration of GC cells. Mechanically, HOXD9 directly associated with the RUFY3 promoter to increase the transcriptional activity of RUFY3. Inhibition of RUFY3 attenuated the proliferation, migration and invasiveness of HOXD9-overexpressing GC cells in vitro and in vivo. Moreover, both HOXD9 and RUFY3 were highly expressed in cancer cells but not in normal gastric tissues, with their expressions being positively correlated.

**Conclusions:**

The evidence presented here suggests that the HOXD9-RUFY3 axis promotes the development and progression of human GC.

**Electronic supplementary material:**

The online version of this article (10.1186/s13046-019-1399-1) contains supplementary material, which is available to authorized users.

## Introduction

Homeobox (HOX) genes encode a highly conserved family of transcription factors that significantly influence many cellular processes, including proliferation [1], apoptosis [2], cell shape [3], and cell migration [4]. HOX genes contain a conserved 183 bp sequence and encode nuclear proteins called homeoproteins [5, 6]. HOX proteins regulate the expression of many downstream target genes. Novel evidence has confirmed the involvement of the dysregulated expression of HOX genes in cancer [4–6]. HOX genes are a family of 39 transcription factors that are divided into four clusters (HOXA to HOXD); during normal development, these genes regulate cell proliferation and specific cell fates [7] .

The transcription factor HOXD9 is one of the members of the HOX family, which has emerged as a family of important transcriptional regulators [8–10]. HOXD9 participates in the development and progression of some cancers. Studies have demonstrated that HOXD9 contributes to both cell proliferation and/or cell survival in gliomas [8]. Moreover, the HOXD9 expression level was higher in esophageal cancer tissues than that in normal tissues [9]. Furthermore, HOXD9 exhibited high expression in invasive HCC cells, and HOXD9 overexpression can significantly enhance HCC cell migration, invasion, and metastasis [10]. The transcription factor HOXD9 binds to a DNA consensus sequence (5′- AATAAAAATA − 3′) (http://alggen.lsi.upc.es/cgi-bin/promo_v3/promo/promoinit.cgi?dirDB=TF_8.3) to regulate transcription, and it can regulate ZEB1 expression [10]. These findings emphasize that HOXD9 may play a crucial role in endowing tumor cell malignant behavior.

Human RUFY3 (RUN and FYVE domain containing 3), also known as RIPX (Rap2 interacting protein X) or Singar1 (singleaxon-related1), is highly expressed in brain tissue [11]. The N-terminal region of RUFY3 and its homologs, including RPIP8 and RPIP9, contain the RUN domain, which can interact with Rap2 and Rab. The crystal structures indicate that RUFY3 contains a RUN domain and two coiled-coil domains [12]. RUFY3 appears to play an important role in multiple Ras-like GTPase tumor pathways [13]. We and others have implicated RUFY3 as an oncogenic factor, and RUFY3 induces gastrointestinal cancer cell migration [14, 15]. We also showed that RUFY3 physically interacts with FOXK1 and the RUFY3-FOXK1 axis, which might promote the development and progression of human GC [16]. We analyzed the RUFY3 interaction with the proximal promoter using Promo software and may have found a HOXD9 sequence-specific binding site. However, the mechanism by which HOXD9 regulates RUFY3 expression through transcriptional activation to promote cell growth, invasion and metastasis needs further investigation.

In this study, we sought to determine the role of HOXD9 in the growth, migration and invasion of GC. We identified that HOXD9 directly regulates RUFY3 expression in GC and sequentially promotes tumor proliferation, metastasis and invasion. This important HOXD9-RUFY3 signaling pathway may be used for future targeted therapy of GC.

## Materials and methods

### Cells, antibodies and reagents

An immortalized normal gastric epithelial cell line GES-1, human gastric carcinoma cell line SGC-7901 and BGC-823 and MGC-803 (Beijing Institute of Cancer Research, Beijing, China), AGS and HGC-27 (ATCC, Rockville, MD), MKN-45 and MKN-28 (Japanese Collection of Research Bioresources Cell Bank, Osaka, Japan) were obtained. Cell lines were maintained at 37 °C in a 5% CO_2_ at mosphere in PRMI-1640 medium containing10% heat-inactivated fetal bovine serum with penicillin and streptomycin.

Rabbit antibodies against Ki-67 (ab155890–100) and CD105 (ab169545) were purchased from abcam (Cambridge, MA, UK). Rabbit anti-HOXD9 (SAB4200029-200UL), anti-Erk1/2 (137F5), p- Erk1/2 (Thr202/Tyr204), anti-p38 (#9212) and p-p38 (#9211), mouse anti-human c-Jun (G-4) and p-JNK (G-7) were purchased from CST (MA, USA). Mouse anti-human FHL2 was purchased from MBL International Incorporation (Woburn, Japan). Rabbit anti-Vimentin (Ag0489), MMP2 (10373–2-AP) and MMP9 (10375–2-AP) were acquired from Proteintech (Wuhan, China), Rabbit anti-RUFY3 (H-140) and horseradish peroxidase-conjugated anti-rabbit, mouse anti-Cyr61(H-78), anti-FOXK1 were purchased from Santa Cruz Biotechnology (Santa Cruz, CA, USA). Mouse anti-β-Tubulin (RM2003) was purchased from Ray antibody Biotech. The specific phospho-ERK inhibitor PD98059, phospho-JNK specific inhibitor SP600125 and phospho-p38 specific inhibitor SB203580 were purchased from Calbiochem (Merck KGaA, Darmstadt, Germany).

### Tissue multi-array (TMA) and immunohistochemical analysis

Immunohistochemical staining on tissue microarray of GC was carried out as described previously [17]. See Additional file 1: Supplementary Materials and Methods.

### Plasmids construction

Normal human complementary DNA (cDNA) corresponding to the full-length HOXD9 was obtained by RT-PCR amplification. The PCR products were subcloned into mammalian expression vector pENTER-FLAG (ViGene Biosciences, Rockville, MD, USA). Populations of pENTER vector and pENTER HOXD9 stable transfectants were obtained using the same plasmid and selection process. Cell transfection was performed with Lipofectamine TM 2000 (Invitrogen) as described in the manufacturer’s protocol.

### RNA isolation, RT-PCR, and real time PCR

Using motorized homogenizer, the snap frozen tissue(~ 100 mg) was ground and total RNA was isolated using Tri-reagent (Sigma Aldrich, St Louis, MO, USA) according to manufacturer’s protocol. cDNA was synthesized from 1 μg of total RNA using Gene-Amp RNA PCR cDNA synthesis kit (Applied Biosystems, Carlsbad, CA,USA). Each cDNA sample was processed in duplicate for each q-RT-PCR assay, and the average relative fold mRNA expression levels were determined using the 2^-ΔΔC^_t_ method, with GAPDH as the internal control. The primers used are listed in Additional file 1: Table S1.

### Western blot analysis

Cell lysates were prepared using lysis buffer with 1%NP40 detergent, 0.5% sodium deoxycholate, 0.1% SDS,50 mM sodium fluoride, 1 mM sodium orthovandate,10 mM sodium pyrophosphate (Sigma Aldrich) and protease inhibitors (Roche). Protein was quantified with Bradford reagent and equal amount of protein was resolved by SDS-PAGE using Bio-Rad apparatus, transferred to PVDF membrane (Millipore, Billerica, MA,USA) and probed with appropriate antibodies. HRP coupled secondary antibodies were obtained, and proteins were visualized using the enhanced chemiluminescence detection system.

### Gene silencing using siRNA

HOXD9 siRNA and Scrambled control siRNA were purchased from Genepharma Company (Suzhou, China). The sequences of the siRNA were as follows (sense strand): HOXD9-specific siRNA (875- CACCAAAUACCAGACGCUU -894); RUFY3 siRNA 1: 1295-GACTAATCAGATGGCTGCTACCA-1318; RUFY3 siRNA 2:194-GGCTAATGAACGCATGAAC-213 and src siRNA, 5′-TTCTCCGAACGTGTCACGT-3′. The siRNA was transfected into the cells with lipofectamine, following the protocols provided by the manufacturer.

### Cell proliferation assay and colony forming assay

See Additional file 1: Supplementary Materials and Methods.

### EdU incorporation assay

See Additional file 1: Supplementary Materials and Methods.

### Invasion and cell migration assays

Invasion and cell migration assays were performed as described [4, 18]. The cells were plated in serum-free medium on Transwell inserts (Corning, NY) coated with 25 μg of Matrigel (BD Biosciences) for invasion assays. After incubation for 48 h at 37 °C/5% CO_2_, the inserts were fixed with 3.7% paraformaldehyde/PBS and stained with 2% crystal violet. The number of cells that had invaded was counted in five representative (**×** 200) fields per insert (invasion index, %). Cell migration assays were performed. Briefly, the cells plated in six well plates with 100% confluence were wounded with a pipette tip at time 0. The media were changed to remove cell debris, and the cells were cultured in the presence of 10 μg/ml mitomycin C to inhibit cell proliferation. Photographs were taken 72 h later.

### Gelatin zymography assay

The production of MMPs (MMP2 and MMP9) in GC cells was analyzed by gelatin zymography. GC cells were grown to 70% confluence, washed twice with 1 × PBS, and incubated in serum-free medium. After 24 h, the conditioned medium was collected and concentrated with a centrifugal filter (Millipore, MA, USA) under 6000 *g* for 15 min. Gelatin zymography assays were performed using commercial kits (Novex 10% Gelatin Gel, Invitrogen). The gel was stained with Coomassie blue. Densitometry was used to quantify the MMP bands.

### Luciferase assay

First, 104-bp (RUFY3p1) and 345-bp (RUFY3p2) fragments of the RUFY1 promoter upstream of the transcription start site were cloned into the pGL3basic vector. For the luciferase assay, the cells were transiently transfected with the various pLuc constructs with Lipofectamine 2000 (Invitrogen, Carlsbad, CA, USA). Luciferase activity was measured sequentially from a single sample using the Dual-Glo™ Luciferase Assay System (Promega) as described previously [19]. The firefly luciferase activity was normalized against Renilla activity, and the relative amount of luciferase activity in the untreated cells was designated as 1. The luminescence was measured with a dual luminometer (TD-20/20, EG&G, Berthold, Australia).

The mutant RUFY3 promoter reporter construct was generated from the RUFY3p1 and RUFY3p2 constructs by using the QuikChange site-directed mutagenesis kit (Stratagene, La Jolla, CA). All mutations were verified by sequencing. The primer sequences are listed in the Additional file 1: Table S1.

### ChIP assay

See Additional file 1: Supplementary Materials and Methods. The primers and antibodies used in the ChIP assays are listed in Additional file 1: Table S1.

### Lentivirus preparation

Lentivirus expressing EGFP/HOXD9 (LV-HOXD9) was constructed by Genechem (Shanghai, China) using Ubi-MCS-3FLAG-CBh-gcGFP-IRES-puromycin vector. Ubi-MCS-3FLAG-CBh-gcGFP-IRES-puromycin empty vectors were used as controls (Shanghai Genechem Co. Ltd., China). Double-stranded oligonucleotides encoding human RUFY3-vshRNA (NM_001037442: CCGGGACTAATCAGATGGCTGCTACCATCAAGAGTGGTAGCAGCCATCTGATTAGTCTTTTG) were annealed and inserted into the short hairpin RNA (shRNA) expression vector U6-MCS-Ubiquitin-Cherry-IRES-puromycin. Selected pools of overexpressing and knockdown cells were used for subsequent experiments.

### In vivo tumorigenesis in nude mice

A total of 1 × 10^7^ logarithmically growing AGS cells transfected with LV-EGFP/HOXD9 + src-shRNA, LV- EGFP/HOXD9 + RUFY3-shRNA) and the control LV-EGFP/vector (*N* = 3) in 0.1 ml RPMI 1640 medium were subcutaneously injected into the left-right symmetric flank of 4–6-week-old male BALB/c nu/nu mice. The animals were fed with an autoclaved laboratory rodent diet. Tumors were measured with calipers every 3–5 days after injection, and the tumor volumes were calculated according to the following formula: 0.5 × length × width^2^. All animal studies were conducted in accordance with the principles and procedures outlined in the Southern Medical University of China Guide for the Care and Use of Animals. After 25 days, the mice were sacrificed. Tumor tissues were excised and weighted.

### In vivo metastasis assay

To investigate the role of RUFY3 in HOXD9-mediated in metastasis in vivo, we have established both tail-vein model and orthotopic implantation model which result in lung or liver metastasis by human GC cells. To assess the effect on lung metastasis, we divided in 3 experimental groups (EGFP/vector, EGFP/HOXD9 + src-shRNA and EGFP/HOXD9 + RUFY3-shRNA in 5 × 10^6^/ml cells) with 3 animals each group and injected via the tail vein. The progression of cancer cell growth was monitored after 42 days by bioluminescent imaging using the IVIS100 Imaging System (Kodak, Rochester, NY, USA).

To evaluate the effect on liver metastasis, we injected subcutaneously into the right flank of nude mice (*N* = 6 per group). Six-eight weeks later, when the size of tumor was around 1 cm^3^, tumor mass from each group was taken out and minced into pieces of approximately 1 mm^3^ for use in transplantation. Then, the stomach was exteriorised through a small midline laparotomy and a piece of tumor tissue sutured to the greater curvature side of the gastric antrum surface with a single Maxon 7/0 suture. After implantation, the abdominal wall was closed in two layers with Dexon 5/0. Mice were sacrificed at 6th post-operative week. Four mM paraffin-embedded sections of lung and liver tissues were prepared. The sections were stained with HE and IHC and examined for the presence of metastatic tumor foci under microscopy.

### Statistical analysis

Statistical analyses were performed using the SPSS statistical software package (standard version 20.0 PSS, Chicago, IL). Quantitative data obtained from experiments with biological replicates are shown as the mean ± standard deviation. The survival rates were calculated by Kaplan-Meier curves, and log-rank tests were used to examine the differences in survival rates between the two groups. Two-tailed Student’s t-test was used to analyze the quantitative data, with significant differences being considered if P values were < 0.05.

## Results

### HOXD9 is overexpressed in gastric cancer tissues and GC cell lines

In the firebrowse database (http://firebrowse.org/), HOXD9 is upregulated in gastric (STAD), stomach and esophageal (STES), thyroid (THCA), bladder urothelial (BLCA), bile duct (CHOL), esophageal (ESCA), head and neck (HNSC), hepatocellular (LIHC), lung adenocarcinoma (LUAD) and lung squamous (LUSC) cancer tissues compared with that in corresponding normal tissues (Additional file 2: Figure S1). This finding suggests that HOXD9 may be associated with various types of cancer, including gastric cancer (GC).

**Fig. 1:**
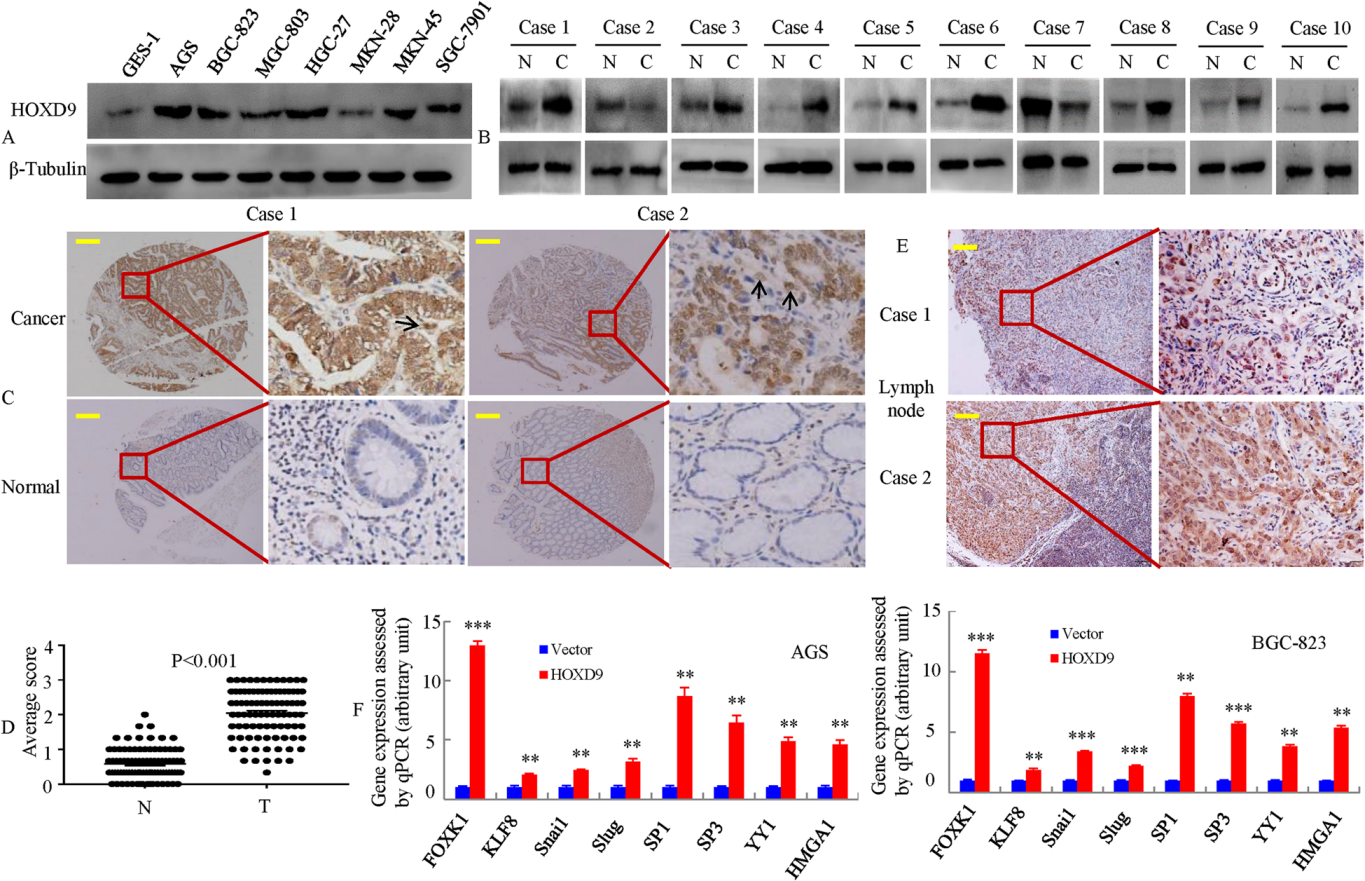
HOXD9 is overexpressed in GC cell lines and tissues. **a** Whole lysates of GES-1, AGS, BGC-823, MGC-803, HGC-27, MKN-28, MKN-45 and SGC-7901 were collected, and HOXD9 was detected by Western blot. β-tubulin was used as the internal control. **b** The expression of HOXD9 by Western blot in 10 pairs of GC and matched noncancerous gastric tissues. N: normal; C: cancer. Arrow, tumor-associated stroma cells (TAS) (**c**) HOXD9 expression in normal and malignant human gastric tissues was detected by TMA. **d** Expression of HOXD9 was determined in normal and cancerous gastric tissues. ****, *P* < 0.001. **e** Representative IHC images are shown for HOXD9 expression in lymph node metastatic cancer tissues. **f** Expression of multiple transcription factor oncogenes in transient transfection as detected by qRT-PCR in GC cells. *, *P* > 0.05, **, *P* < 0.05, ***, *P* < 0.01. Scale bars, 100 μm in **c** & **e**

Next, we examined the expression of HOXD9 in the following seven gastric cancer cell lines: AGS, BGC-823, MGC-803, HGC-27, MKN-28, MKN-45, SGC-7901 and immortalized gastric mucosal epithelial cell line GES-1. As shown in Fig. 1a, the expression of the HOXD9 protein level in gastric cancer cells was significantly higher than the expression of GES-1.

We then measured HOXD9 expression in matched gastric normal (N) and cancerous (T) tissues by Western blot. We proved that eight of ten samples of GC specimens showed higher expression of HOXD9 protein than adjacent normal tissues (Fig. 1b).

To further evaluate the effect of HOXD9 expression on GC progression and metastasis, we investigated the expression of HOXD9 protein with a 90-primary GC tissue microarray (TMA). We observed HOXD9-positive staining in tumor cells and tumor-associated stroma cells (TAS; arrow) and HOXD9-negative or weak HOXD9-positive staining in adjacent normal gastric cells as exemplified in Fig. 1c. Semiquantitative scoring showed that HOXD9 protein was expressed at significantly higher levels in cancer tissues than that in adjacent normal gastric mucosa tissues by TMA assay (Fig. 1d).

To validate our findings in vivo, we examined HOXD9 expression in regional lymph nodes and metastatic cancer tissues from two patients (Fig. 1e). HOXD9 was expressed at high or intermediate levels in cancer cells. These results suggest that HOXD9 is upregulated in human GC.

Finally, we transfected the GC cells with empty (vector) plasmid and plasmids encoding HOXD9 protein into GC cells. After selection with antibiotics, we obtained a pooled cloned cell line that stably expressed HOXD9. We confirmed the induced expression of eight transcription factor oncogenes after ectopic HOXD9 expression in GC cells. The mRNA expression of the endogenous FOXK1, KLF8, Snail, Slug, Sp1, Sp3, YY1 and HMGA1 genes were upregulated in stable HOXD9 transfectants of GC cells (Fig. 1f).

These results support the hypothesis that HOXD9 might play a role in the tumorigenesis of gastric cancer.

### HOXD9 expression is associated with pathologic features and poor prognosis of GC patients

Based on the data from TMA, we analyzed the correlation between high HOXD9 expression and the clinicopathological features of GC; the data are summarized in Table 1. We showed that increased HOXD9 expression is correlated with differentiation (*P* < 0.001), lymph node metastasis (*P* < 0.001), tumor size (<10 cm^3^ vs ≥ 10 cm^3^, *P* = 0.001), AJCC stage (I/II vs. III/IV, *P* = 0.001) and TNM stage (I/II vs. III/IV, *P* < 0.001). However, no significant association was observed between HOXD9 expression and age (<60 y vs ≥60 y, *P* = 0.475) or sex (*P* = 0.294).

**Table 1:** Correlation between HOXD9 protein expression and the clinicopathological parameters of gastric carcinoma

Features	Total number (*n* = 90)	HOXD9 Expression		*P*
		Low	High	
Age (years)				
<60	35	12 (34.3%)	23 (65.7%)	0.475
≥60	55	23 (41.8%)	32 (58.2%)	
Gender				
Male	53	23 (43.3%)	30 (56.6%)	0.294
Female	37	12 (32.4%)	25 (67.6%)	
Differentiation				
Well	29	22 (75.9%)	7 (24.1%)	<0.001
Moderate	17	8 (47.1%)	9 (52.9%)	
Poor	44	5 (5.6%)	39 (88.6%)	
Lymph node metastasis				
Yes	67	15 (22.4%)	52 (77.6%)	<0.001
No	23	20 (87.0%)	3 (13.0%)	
Tumor size (cm^3^)				
<10	24	16 (66.7%)	8 (33.3%)	0.001
≥10	66	19 (28.8%)	47 (52.2%)	
AJCC stage				
T1, T2	14	11 (78.6%)	3 (21.4%)	0.001
T3, T4	76	24 (31.6%)	52 (68.4%)	
AJCC TNM stage				
I, II	36	27 (75.0%)	9 (25.0%)	<0.001
III, IV	54	8 (14.8%)	46 (85.2%)	

**Fig. 2:**
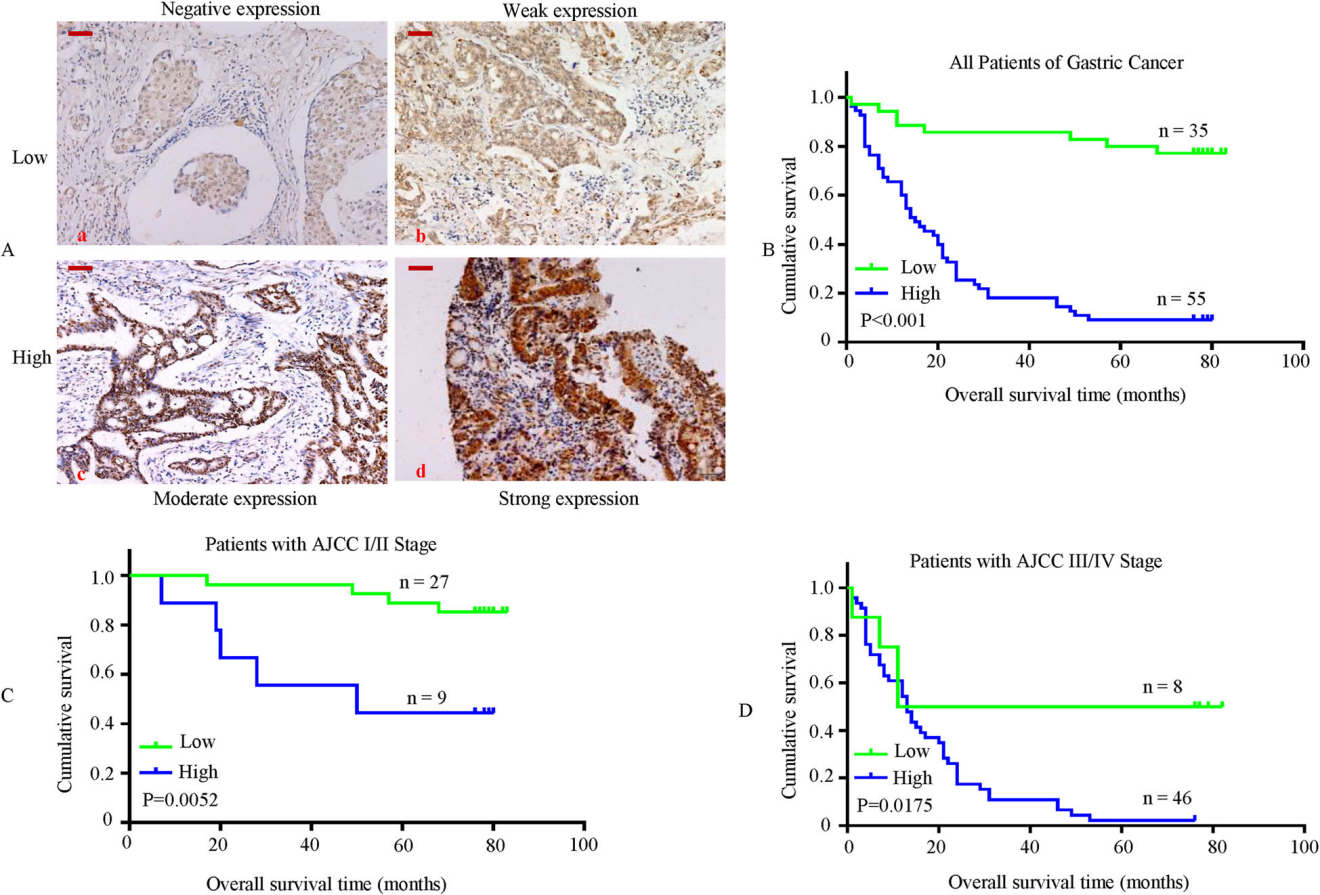
HOXD9 is upregulated in gastric cancer tissues and associated with poor prognosis. **a** TMA representative images of IHC staining of HOXD9 in GC tissue. **a** Negative HOXD9 expression; (**b**) weak HOXD9 expression; (**c**) moderate HOXD9 expression; (**d**) strong HOXD9 expression in GC. Scale bars, 50 mm in A. Kaplan–Meier survival analysis of overall survival in all patients (**b**), the patients at the early stage of GC (**c**) and the patients at the late stage of GC (**d**) according to HOXD9 expression. The log-rank test was used to calculate *P*-values

Moreover, we assessed the prognostic effect of HOXD9 on overall survival by comparing the overall survival of GC patients with high or low HOXD9 protein levels (Fig. 2a). Of the 90 surgical GC specimens, 55 cases showed high HOXD9 expression, whereas low expression was found in the other 35 cases. The Kaplan-Meier survival curves demonstrated that GC patients with high HOXD9 expression had a significantly poorer 80-month survival rate than those with low HOXD9 expression (Fig. 2b). Such a relationship was observed in patients with late-stage GC (AJCC stage III and IV) (*P* = 0.0175, Fig. 2d), but it was less obvious in those with early-stage GC (AJCC stage I and II) (*P* = 0.0052) (Fig. 2c). Similar to the results from the KM-Plotter database (http://kmplot.com/analysis/index.php?p=service&start=1) (Additional file 3: Figure S2A) and TCGA dataset (http://xena.ucsc.edu/public, Additional file 3: Figure S2B), there was a significant difference between the HOXD9 high-expression group and the HOXD9 low-expression group. High-expression of HOXD9 gene in overall survival (OS) of GC patients predicts poor prognosis than low-expression HOXD gene at mRNA level (Additional file 3: Figure S2A & B).

These findings indicate that HOXD9 expression plays a critical role in GC development and progression and is a valuable prognostic biomarker for this disease.

### The expression of HOXD9 is correlated with the malignant biological behavior in GC

**Fig. 3:**
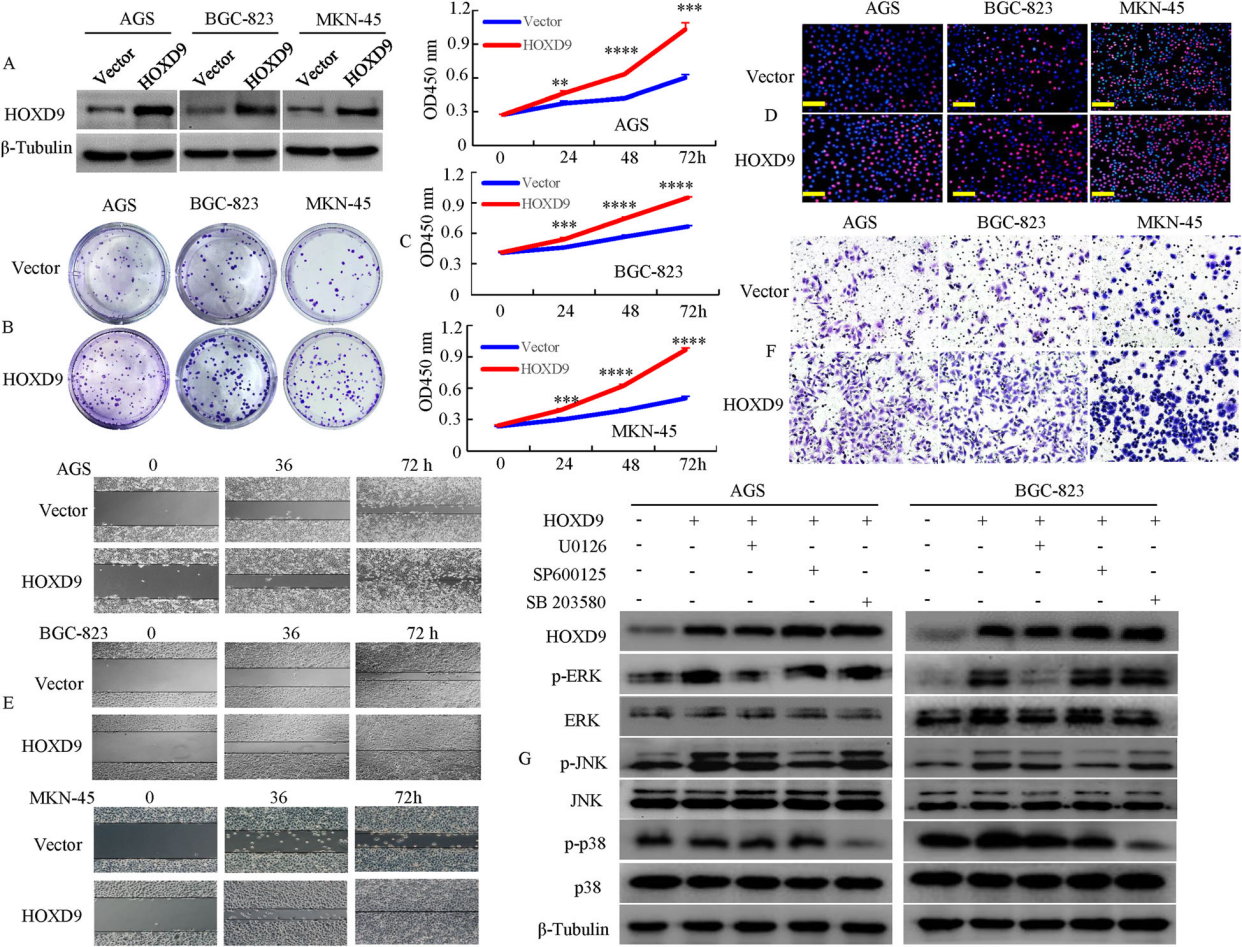
The overexpression of HOXD9 is correlated with malignant biological behavior in GC. **a** HOXD9 expression was detected by western blot with β-tubulin used as the internal control. **b** Proliferation in GC cells transfected with vector or HOXD9 was assessed by the colony-forming assay. **c** Proliferation of GC cells was assessed by CCK-8 assay. **, *P* < 0.05, ***, *P* < 0.01, ****, *P* < 0.001. **d** DNA synthesis in GC cells was measured by the EdU incorporation assay at 48 h after the indicated transfection. Red fluorescence represents the EdU-positive cells; blue fluorescence is from the Hoechst 33342 stain and represents the total cells. **e** Scratch wounds were made in monolayer cultures of cells after plating. A quicker closure of the wound in the HOXD9 cells culture from 0 to 72 h based on the wound width is shown in the graph compared to the controls. **f** Tumor cell invasion capacity was measured with Transwell chambers. Representative image fields of invasive cells on the membrane are shown. **g** The GC cells were treated with U0126 (20 μM), SP600125 (20 μM) or SB203580 (3 μM) for 48 h. Gene expression was detected by Western blot analysis. β-tubulin was used as the internal control. Scale bars, 100 mm in **d**

Given the increased expression of HOXD9 in the GC tissues, we evaluated the functional effect of HOXD9 overexpression on cell behaviors in vitro. We first established pooled- stable transfectants with HOXD9-sense and vector plasmids to verify the protein expression levels. The results indicated that overexpression of exogenous HOXD9 were confirmed by western blot analysis the GC cell lines (Fig. 3a). Subsequently, GC cells proliferation were evaluated. As illustrated in Fig. 3b and Additional file 4: Figure S3A, HOXD9 overexpression resulted in an increased number of colonies compared with cells transfected with empty vector in a colony forming assay. The forced expression of HOXD9 significantly enhanced GC cellular growth rates, with the highest increased peak at 72 h at the determined time periods in this study compared to those of empty vectors using the CCK-8 assay (Fig. 3c). Similarly, the proliferation rate of GC cells transfected with HOXD9 was significantly increased compared to that of the empty vector by using an EdU assay (Fig. 3d and Additional file 4: Figure S3B). These results indicate that HOXD9 overexpression can lead to increased GC cell growth.

Lv X et al. have demonstrated that HOXD9 promotes the migration and invasion of liver cancer cells [10]. Motivated by this previous study, we studied the effect of HOXD9 on the migration and invasion of GC cells. As shown in Fig. 3e, the forced expression of HOXD9 significantly enhanced the migratory capacity of GC cells (Fig. 3e and Additional file 4: Figure S3C). Moreover, we examined its cell invasion activity in vitro. After ectopic expression of HOXD9, the invasiveness of GC cells was increased compared with that of control cells (Fig. 3f and Additional file 4: Figure S3D).

A previous report showed that most cancers are associated with deregulation of the MAPK/ERK (ERK, JNK, and p38) signaling pathways [20]; thus, we explored the mechanism of HOXD9’s effect on proliferation and migration in GC cells. The ectopic expression of HOXD9 enhanced the phosphorylation levels of extracellular signal-regulated protein kinase (ERK1/2) and c-Jun N-terminal kinase (JNK). However, the overexpression of HOXD9 was associated with unchanged phosphorylation levels of p38 mitogen-activated protein kinases (p38) by Western blot (Fig. 3g). Then, we treated the cells with the ERK1/2 inhibitor U0126, the JNK inhibitor SP600125 or the p38 inhibitor SB203580 for 2 days. Inhibitor U0126, SP600125 or SB203580 treatment inhibited the activation of p-ERK, p-JNK and p-p38, but ERK, JNK and p38 levels remained unchanged.

These studies suggest that HOXD9 promotes the proliferation and invasive capacity of GC cells.

### RUFY3 is a direct target of transcriptional activation by HOXD9

**Fig. 4:**
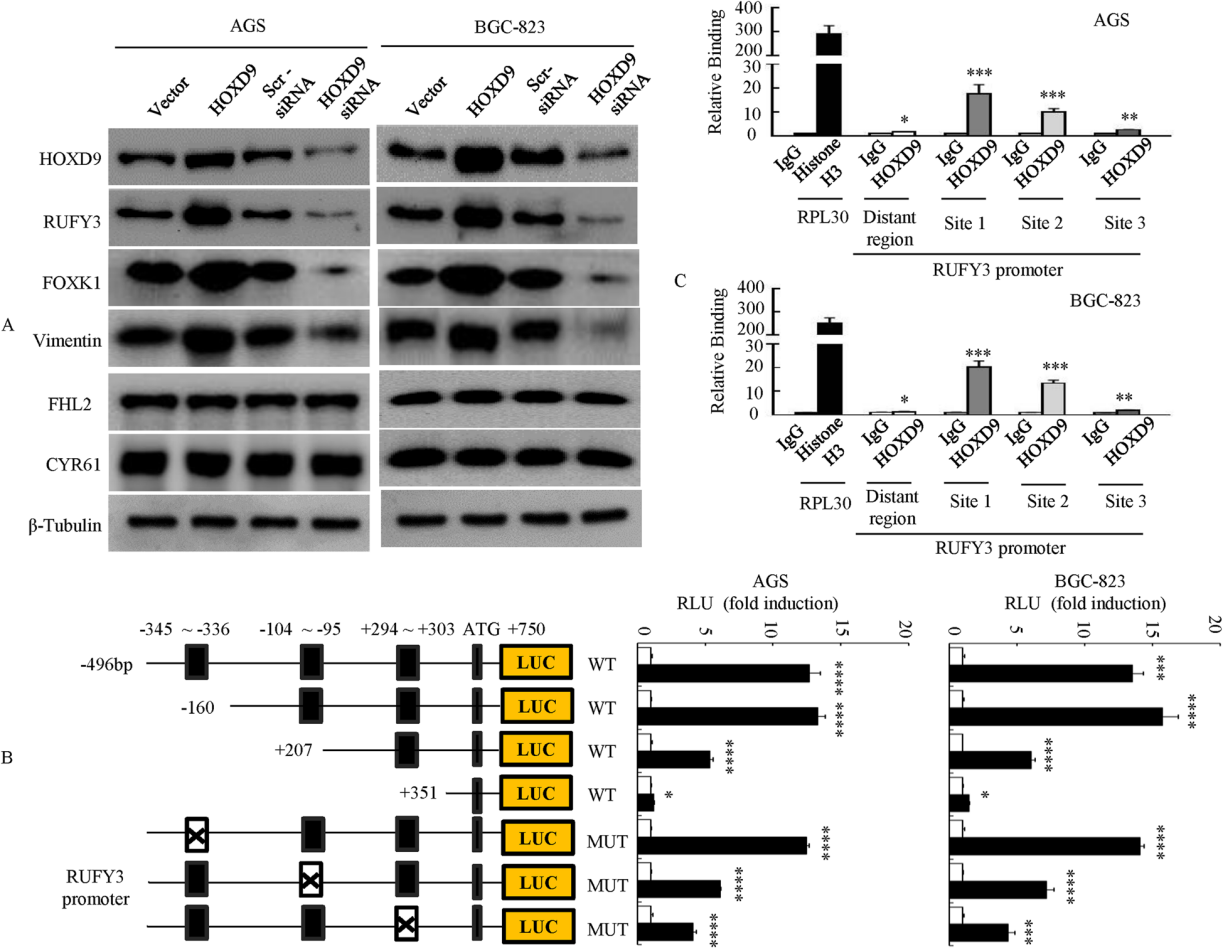
HOXD9 regulates RUFY3 transcriptional activity by binding to the RUFY3 promoter. **a** The vector and HOXD9 plasmid, or Src siRNA and HOXD9 siRNA, were transfected into GC cells. The HOXD9, RUFY3, FOXK1, Vimentin, FHL2 and Cyr61 expression levels were detected in GC cell lines using Western blot analysis. **b** Deletion or selective mutation analysis identified a responsive transcription factor HOXD9 binding site in the RUFY3 promoter. Truncated and mutated RUFY3 promoter constructs were cotransfected with HOXD9, and relative luciferase activities were determined. ■, wildtype site and □, mutated site. *, P > 0.05, ***, P < 0.01 and ****, P < 0.001. **c** A ChIP -qPCR assay demonstrated the direct binding of HOXD9 to the RUFY3 promoter in GC cells. Gene enrichment was quantified relative to input controls by qPCR using primers specific for the promoter regions of RUFY3. Results are shown as a fold change of qPCR value over IgG. *P* > 0.05, **, *P* < 0.05 and ***, *P* < 0.01

We previously reported that genes such as Cyr61, FHL2, Vimentin, FOXK1 and RUFY3 are implicated in the pathogenesis of gastrointestinal cancers [14, 17, 18, 21, 22]. As a transcription factor, HOXD9 may regulate downstream genes; thus, we further assessed whether the overexpression or knockdown of HOXD9 modulated the expression of a panel of genes (Cyr61, FHL2, Vimentin, FOXK1 and RUFY3) in GC cells. We showed that the upregulation of HOXD9 significantly increased the expression of FOXK1, Vimentin and RUFY3, whereas the downregulation of HOXD9 decreased FOXK1, Vimentin and RUFY3 expression. In contrast, the protein expression level of FHL2 and Cyr61 remained unchanged upon HOXD9 overexpression or knockdown (Fig. 4a). Therefore, we wondered whether the transcription factor HOXD9 could directly regulate RUFY3 in GC cells.

We studied the interaction between RUFY3 and HOXD9 proteins, and there was no association using the STRING database (https://string-db.org/cgi/input.pl). We next assessed whether HOXD9 proteins could directly bind to the RUFY3 promoter.

We scanned the proximal 1250 bp of the promoter region of RUFY3 using Promo software (http://alggen.lsi.upc.es/cgi-bin/promo_v3/promo/ promoinit.cgi?dirDB = TF_8.3) and identified the following three potential HOXD9 binding sites: site 1 (RUFY3p1), + 294 ~ + 303; and site 2 (RUFY3p2), − 104 to − 95; and site 3 (RUFY3p3), − 345 to − 336 (numbering is from the transcription start site; Fig. 4b). The luciferase reporter assay showed that the activity of RUFY3p1 and RUFY3p2 in HOXD9 cells increased approximately 5 ~ 14-fold in AGS and 6 ~ 15 fold in BGC-823 cells compared with that in vector control cells, whereas the magnification exhibited a slight decrease with RUFY3p3. Site-directed mutagenesis showed that the first and second HOXD9-binding site were critical for HOXD9-induced RUFY3 transactivation (Fig. 4b).

Next to investigate whether HOXD9 could physically bind to the RUFY3 promoter in vivo, we performed chromatin immunoprecipitation (ChIP) -qPCR assays in human tumor cell lines expressing endogenous HOXD9. PCR primers located in exon 3 of RPL30 and anti-Histone H3 antibody served as the positive control. The primers were used to amplify the sequence containing the distant upstream of RUFY3 promoter served as the negative control. qPCR amplification showed that the first (+ 294 to + 303) and the second (− 104 to − 95) possible binding sites were immunoprecipitated. Normal rabbit IgG was used as the negative control. No bands were evident in the immunoprecipitates for the third (− 345 ~ − 336) possible binding site for the control IgG (Fig. 4c). These studies suggest that HOXD9 transactivates RUFY3 expression.

### RUFY3 promotes GC development and progression by regulating HOXD9

**Fig. 5:**
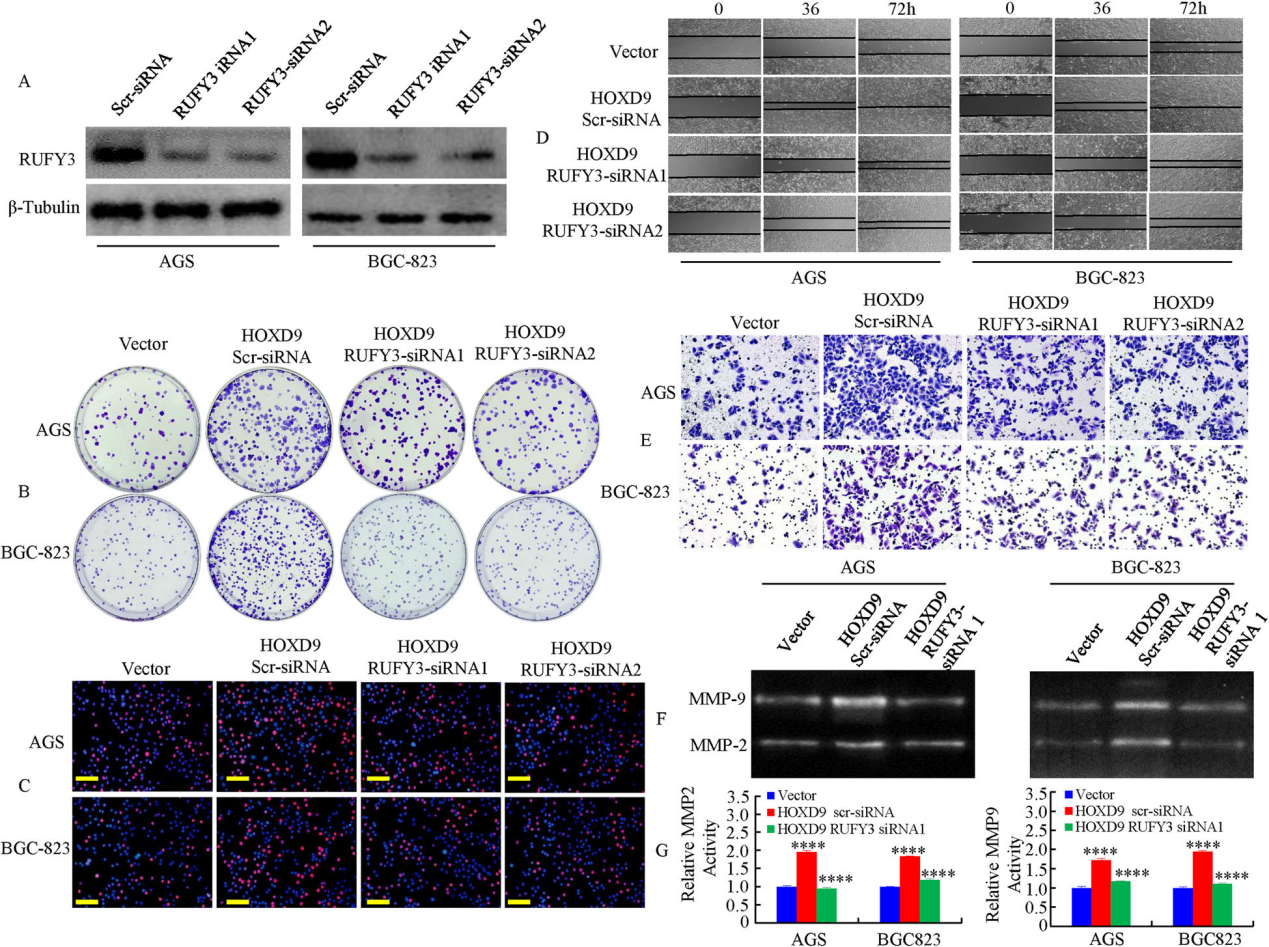
HOXD9-RUFY3 axis promotes the growth and invasion of GC cells in vitro. **a** Expression levels of HOXD9 were detected by Western blot analysis in AGS cells, which were transfected with RUFY3 siRNA1, RUFY3 siRNA2 or scr siRNA (scrambled siRNA) as a negative control. **b** Soft agar colony formation assays using the indicated cell clones. **c** Stable transfectants of HOXD9 after transfection with RUFY3 siRNA1, RUFY3 siRNA2 or scr siRNA for 48 h were subjected to an EdU incorporation assay. **d** & **e** The GC cell migration and invasion assays were performed. **f** & **g** Representative gelatin zymography showing MMP2 and MMP9 activities in GC cells. **g** Quantitative analysis of (**f**). Bars represent the value of intensity of the bright band and are used for statistical analysis. *N* = 3, ****, *P* < 0.001 vector vs. HOXD9 and HOXD9 vs. HOXD9-RUFY3 siRNA, respectively. Scale bars, 100 μm in **c**

To demonstrate whether RUFY3 is required for HOXD9’s effect on the development and progression of GC, we designed two siRNAs (RUFY3 siRNA1 and RUFY3 siRNA2) with different sequences for the RUFY3 RNAi experiments, and both of these successfully suppressed the expression of RUFY3 (Fig. 5a). Then, we revealed that the the downregulation of RUFY3 in HOXD9-overexpressing cells (HOXD9-RUFY3-siRNA1 and HOXD9-RUFY3-siRNA2) caused a decrease in the proliferation of GC cells (Fig. 5b, c and Additional file 5: Figure S4A & B).

Similar results were observed, where the induction of metastatic phenotypes caused by the overexpression of HOXD9 was abolished by the repression of RUFY3, as shown by migration and invasion assays (Fig. 5d and Additional file 5: Figure S4C, Fig. 5e and Additional file 5: Figure S4D).

We also tested the activities of MMP2 and MMP9 in the vector cells as well as the overexpression of HOXD9 and the knockdown of RUFY3 in HOXD9-overexpressing GC cells. We found that HOXD9 overexpression in GC cells significantly elevated the activities of MMP2 and MMP9, as indicated via gelatinolytic assays, compared with that in empty-vector-expressing cells, whereas RUFY3 knockdown in HOXD9-overexpressing cells led to a loss of the enzymatic activities of MMP2 and MMP9 (Fig. 5f & g). Taken together, these results demonstrate that the effect of RUFY3 on the cell proliferation, migration and invasion of GC cells is caused by the expression of HOXD9.

### RUFY3 is essential for the induction of tumorigenesis and metastasis by HOXD9 in vivo

**Fig. 6:**
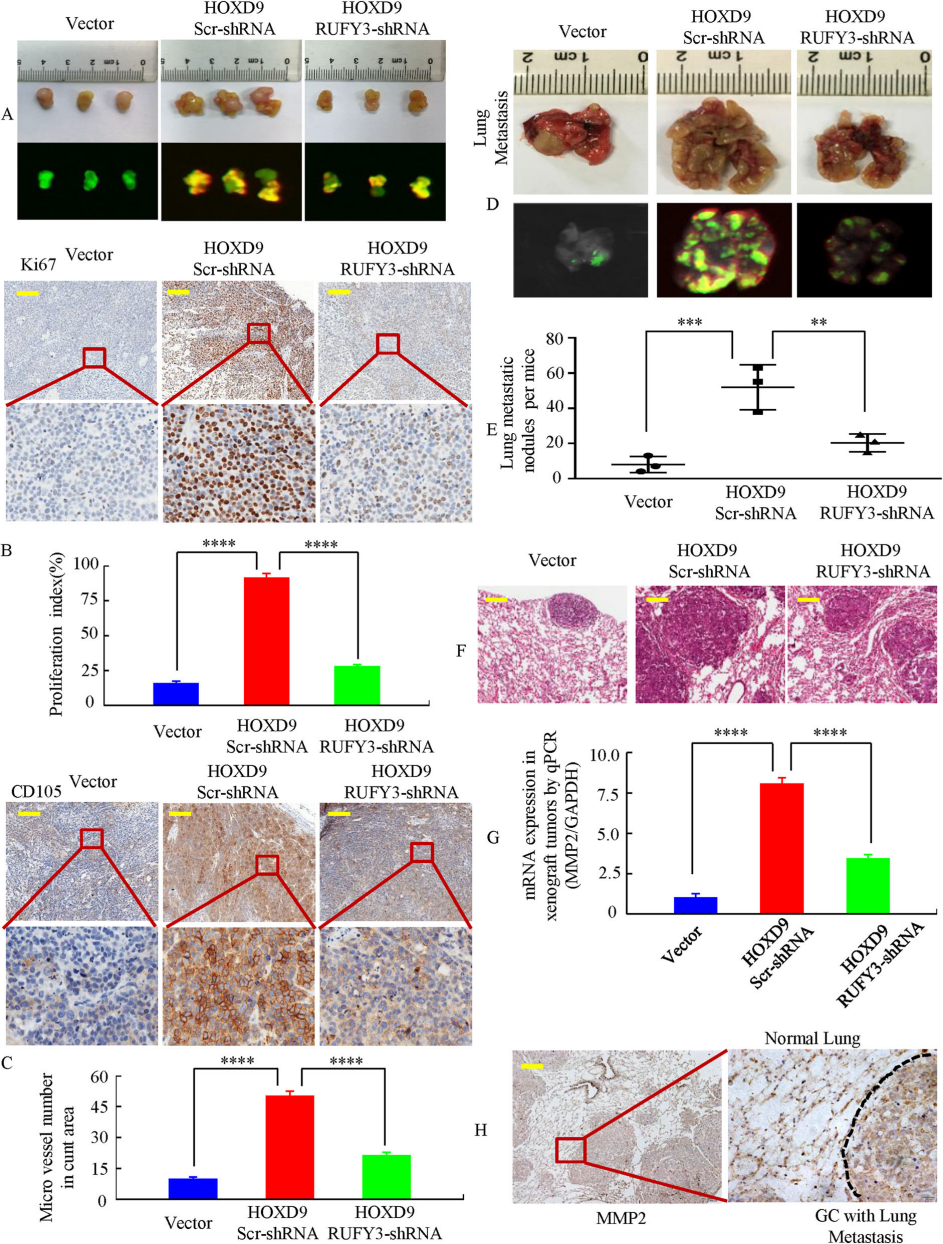
RUFY3 facilitates HOXD9-mediated cell proliferation and metastasis in GC in vivo. **a** External whole-body fluorescence images after subcutaneous injection of AGS/Vector, AGS/HOXD9-scr-shRNA and AGS/HOXD9-RUFY3-shRNA were obtained. The mice were sacrificed. **b** & **c** RUFY3 knockdown significantly inhibited HOXD9-induced proliferation (Ki-67, ****, *P* < 0.001, vector vs. HOXD9 and HOXD9 src-shRNA vs HOXD9-RUFY3-shRNA, respectively), and a considerable decrease of tumor vessel density (CD105, ****, *P* < 0.001, vector vs. HOXD9 and HOXD9 src-shRNA vs. HOXD9-RUFY3-shRNA, respectively) was observed by IHC. **d** Representative images of metastatic loci in the lungs are shown. **e** The number of metastatic loci in the lungs were counted. ***, *P* < 0.01, vector vs. HOXD9; **, *P* < 0.05, HOXD9 src-shRNA vs. HOXD9-RUFY3-shRNA, respectively. **f** Metastatic cancer tissues were stained with H&E. **g** & **h** MMP2 expression in tumors derived from AGS cells was determined by qRT-PCR and IHC; ****, *P* < 0.001, vector vs. HOXD9 and HOXD9 src-shRNA vs. HOXD9-RUFY3-shRNA, respectively. Scale bars, 100 μm in **b**, **c**, **f** and **h**

To verify whether RUFY3 is required for HOXD9 in the growth and metastasis of GC cells, we deployed a RUFY3 loss-of-function strategy in HOXD9-expressing AGS cells in vivo. The cells stably expressing LV-vector, LV-HOXD9 src-shRNA or LV-HOXD9-RUFY3-shRNA in three groups were injected subcutaneously into the right flank of each nude mouse, as shown in Fig. 6a and Additional file 6: Figure S5A & B. The tumor volumes of the HOXD9-overexpressing cells were markedly greater than those of the vector-expressing cells. In contrast, tumors derived from RUFY3 downregulation in HOXD9-overexpressing cells were markedly smaller than those of the vector-treated mice at 20 to 25 days (Additional file 6: Figure S5C & D).

Next, we examined the protein expression of cell proliferation (Ki-67) and angiogenesis (CD105) markers in the xenograft tumor. LV-HOXD9 cell groups was increased compared to that in LV-vector cells, whereas RUFY3 knockdown decreased the proliferation rate and tumor vessel density in the HOXD9-overexpressing group (Fig. 6b & c).

To test the role of RUFY3 in HOXD9-mediated tumor progression, we have established tail-vein metastasis model and orthotropic implantation model using human gastric cancer AGS cells in nude mice, which resulted in lung and liver metastases (Fig. 6d and Additional file 6: Figure S5E & F). he number of lung or hepatic metastatic lesions in mice injected with LV-HOXD9 cells was increased compared to LV-vector cells, whereas knockdown of RUFY3 in HOXD9-overexpressing cells decreased the number of metastatic loci in HOXD9-overexpressing cells (Fig. 6e and Additional file 6: Figure S5G). The presence of metastasis from GC to the lung and liver was confirmed by histological analysis (Fig. 6f and Additional file 6: Figure S5H). We next examined the expression of cell metastasis markers (MMP-2 and MMP-9) by qPCR and IHC. HOXD9-overexpressing group resulted in a significant increase in MMP2 and MMP9 levels, whereas downregulation of RUFY3 in HOXD9-overexpressing group caused a decrease in MMP2 and MMP9 levels in GC tissues in xenograft tumors. (Fig. 6g and Additional file 6: Figure S5I, J & K). MMP2 and MMP9 were expressed at high levels in GC tissues than normal tissues in nude mice (Fig. 6h and Additional file 6: Figure S5L, M & N).

Taken together, these results indicate that the HOXD9-RUFY3 axis contributes to progression and metastasis in vivo.

### HOXD9 expression positively correlates with RUFY3 expression in GC tissue

**Fig. 7:**
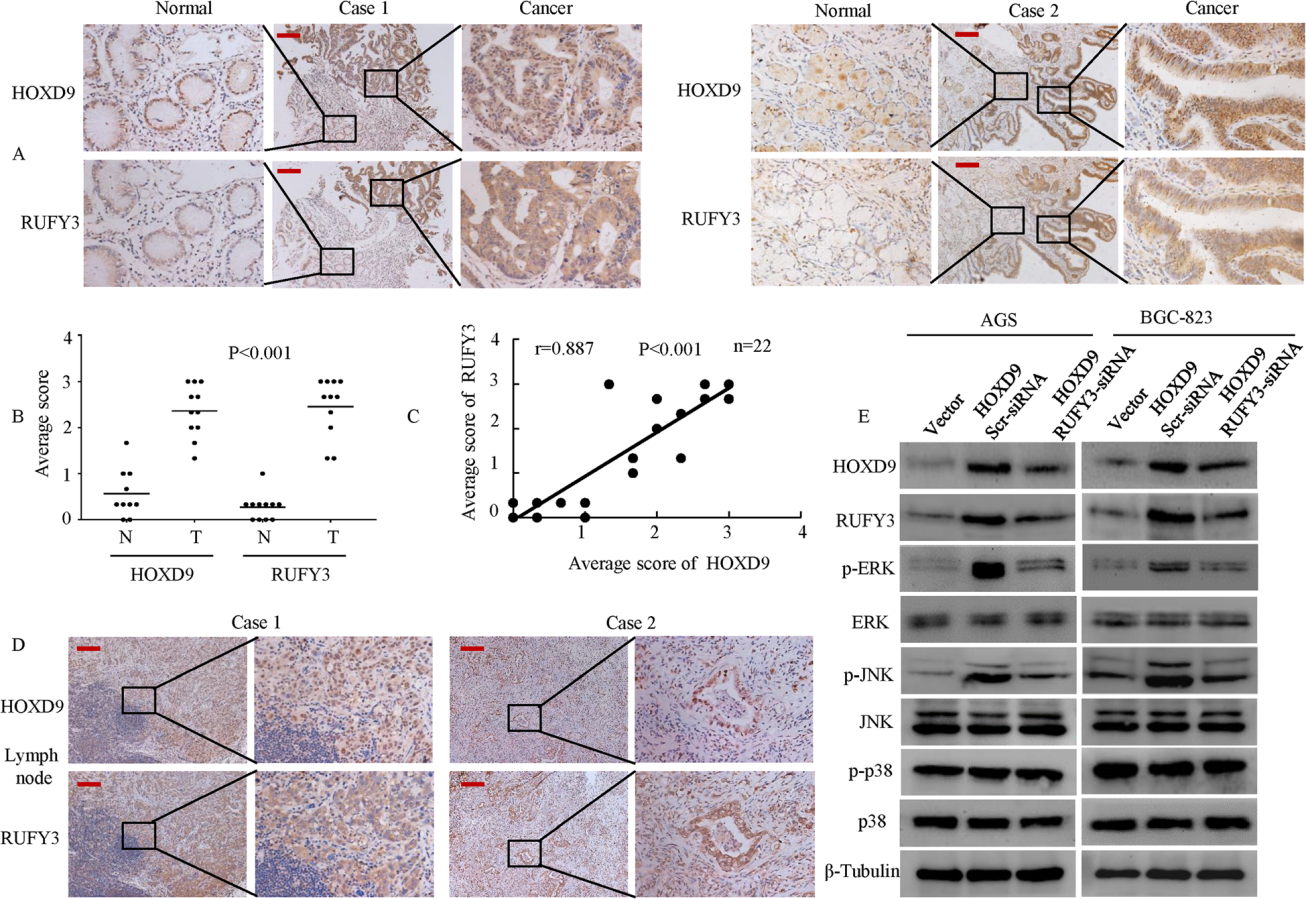
HOXD9 expression is positively correlated with RUFY3 expression in human GC tissues. **a** IHC staining analysis of HOXD9 and RUFY3 expression in 11 samples of GC tissues and adjacent nontumorous tissues. **b** Average scores of the two proteins in normal and cancerous gastric tissues. ****, *P* < 0.001 between normal and cancer tissues. **c** Spearman’s correlation analysis was used to determine the relationship between HOXD9 and RUFY3 protein expression. **d** The representative results of IHC staining for HOXD9 and RUFY3 in serial sections with lymph node metastatic cancer tissues. **e** Western blotting analyzed the expression of the indicated proteins in the GC cells. Scale bars, 200 μm in **a** and **d**

We analyzed both HOXD9 and RUFY3 expression in 11 samples of GC tissues. By analyzing consecutive primary GC sections, we observed that HOXD9 expression was significantly positively correlated with RUFY3 expression. GC but not adjacent tissues expressed both HOXD9 and RUFY3, as exemplified in Fig. 7a. Semiquantitatively scoring the two proteins showed that the expression levels of both proteins in cancerous tissues were significantly higher than those of adjacent normal gastric tissues (Fig. 7b), while Spearman correlation analysis showed a positive correlation between HOXD9 and RUFY3 expression (correlation coefficient r = 0.886, Fig. 7c).

Furthermore, we examined the expression of HOXD9 and RUFY3 in serial sections of lymph node metastatic cancer tissues from two patients. The results showed that a high level of HOXD9 expression positively correlates with RUFY3 expression in lymph node metastases (Fig. 7d).

We had previously shown that activation of the MAPK/ERK pathway is involved in the regulation of cell proliferation, invasion and metastasis in GC (Fig. 3f). We next wondered whether HOXD9 and RUFY3 participated in the MAPK/ERK pathway. The results of Western blots showed that the ectopic expression of HOXD9 led to high phosphorylation levels of p-JNK and p-ERK1/2 compared with that observed in vector cells. However, the p-JNK and p-ERK1/2 phosphorylation levels were downregulated after RUFY3 knockdown in HOXD9-overexpressing cells compared with those in HOXD9-overexpressing cells, and the phosphorylation levels of p-p38 and the total protein levels of JNK, ERK1/2, p38 were unaltered (Fig. 7f).

Together, the data suggest that the expression of HOXD9 is correlated with the expression of RUFY3; thus, the HOXD9-RUFY3 axis has an important role in development and metastasis during GC.

## Discussion

HOX genes, a highly conserved subgroup of the homeobox superfamily, play crucial roles in embryogenesis and tumorigenesis, which share the same requirements such as growth and differentiation. HOX genes that are critical for embryonic development are expressed aberrantly in abnormal development and malignancy, indicating that the altered expression of HOX genes is important for oncogenesis. Numerous examples of aberrant HOX gene expression have been found in various types of cancer, including lung [4], colorectal [23], liver [24] and breast cancers [25]. In the present study, we demonstrated that HOXD9 was upregulated in GC, and patients with a high level of expression of HOXD9 had a poorer prognosis than those with a low level of expression. Furthermore, ectopic expression of HOXD9 in GC cells promoted the proliferation and migration. Moreover, our data showed that HOXD9 transactivated RUFY3 expression. Therefore, HOXD9 may play a critical role in RUFY3-mediated tumor growth and metastasis in vitro and in vivo.

As a member of the HOX family, HOXD9 has been found to be deregulated in several cancers including cervical [26], glioma [9] ovarian [27] and liver cancers [10]. However, the role of HOXD9 in GC is still not well characterized. In this study, we found that HOXD9 was overexpressed in both GC cell lines and tissues.

Kaplan-Meier analysis of the survival curves showed a significantly worse overall survival for patients whose tumors had high HOXD9 levels, indicating that a high HOXD9 tumor protein level is a marker of poor prognosis for patients with GC. Tabuse M et al. showed that HOXD9 promotes glioma cell proliferation, and the knockdown of HOXD9 in glioma cells using HOXD9-specific siRNA resulted in decreased cell proliferation, cell cycle arrest, and the induction of apoptosis [9]. Consistent with this, we showed that HOXD9 promotes GC cell proliferation and that tumors with upregulated HOXD9 tended to display more aggressive behavior. The effect on cell growth, invasion, and metastasis also indicates that HOXD9 functions as an oncogene in cancer cells. All these data suggest that HOXD9 expression is elevated in GC and plays an important role in GC development and progression.

MAPKs are serine/threonine protein kinases, and they can be activated by many stimuli including cytokines [28], growth factors [29], neurotransmitters [30], hormones [31], stress [32], and adhesion [33]. Therefore, MAPKs can be involved in the process of cell division. The MAPK family is divided into three subfamilies: ERK1/2, JNK and p38 [20]; they are involved in many reactions affecting cell proliferation [34], differentiation [35], and apoptosis [36] in mammals. Many studies have confirmed that the MAPK signaling pathway is activated in GC [37], lung cancer [38], ovarian cancer [39], and liver cancer [40]. Therefore, we speculated that HOXD9 might be a downstream factor of MAPK pathways. In this study, we examined the phosphorylation of multiple MAPKs, including ERK1/2, JNK and p38 in GC cell lines. Our results identified important roles for the ERK and JNK signaling pathways in the HOXD9-mediated aggressive behavior of GC cells since there was a dramatic increase in the phosphorylation of ERK and JNK in GC HOXD9-transfected cells compared to that in control vector cells. However, p38 phosphorylation was not changed, suggesting that it is not involved in HOXD9-mediated GC cell metastasis and proliferation. In addition, pretreatment of cells with SP600125 and U0126 resulted in blockade of the increase in HOXD9 expression. Our findings imply that HOXD9 is a positive regulator of the ERK and JNK signaling pathways in vitro.

RUFY is a member of the RUN domain-containing protein family and has been implicated in various biological processes, including embryonic development, cell differentiation, proliferation and apoptosis. It also plays an important role in neoplastic processes such as cell growth, migration and invasion [5, 41, 42]. For example, Zhi Q et al. showed that podocalyxin-like protein promotes gastric cancer progression through interacting with RUFY1 [41]. Wang G et al. also revealed that RUFY3 overexpression promotes gastric cancer cell migration and invasion [5]. However, the roles of HOXD9-RUFY3 axis in GC are still not well characterized. Here, we demonstrated that the forced expression of HOXD9 could also increase the activity of RUFY3 promoter in GC cells, which is in agreement with recent studies, showing that HOXD9 translationally regulates ZEB1 in HCC [10]. Moreover, mutations in the site significantly attenuated the HOXD9-mediated transactivation of the human RUFY3 promoter, and ChIP assays confirmed the recruitment of HOXD9 to the binding site in the human RUFY3 promoter. These studies suggested that RUFY3 was a direct transcriptional target of HOXD9. In addition, abnormal expression of RUFY3 dramatically regulated HOXD9-mediated on the malignant biological behavior of GC in vitro and in vivo. Besides, HOXD9 expression was positively correlated with RUFY3 expression in GC tissue. These results implicated that HOXD9 promotes GC tumorigenesis and metastasis by transactivating RUFY3.

## Conclusions

In summary, we present a novel molecular basis for the role of HOXD9 in GC carcinogenesis, invasion and metastasis. The HOXD9 protein may enhance the malignant properties of tumor cells via its transactivation of the RUFY3 proto-oncogene. Knowledge of the interrelationship between HOXD9 and RUFY3 should greatly advance our understanding of the cellular and molecular mechanisms underlying the biological functions of HOXD9 in GC cells.

## Additional files

Additional file 1: Supplementary Materials and Methods. **Table S1.** Primary Primers Used in This Study. (DOCX 23 kb)

Additional file 2: **Figure S1.** HOXD9 is overexpressed in tumor tissues. The expression pattern of HOXD9 mRNA in normal and tumor tissues. HOXD9 mRNA expression in various types of cancer was searched in the firebrowse database (http://firebrowse.org/). (TIF 3019 kb)

Additional file 3: **Figure S2.** Kaplan-Meier curves for overall survival (OS) from the KM-Plotter database (http://kmplot.com/analysis/index.php?p=service&start=1) (Fig. 2a) and TCGA dataset (http://xena.ucsc.edu/public, Fig. 2b). (TIF 448 kb)

Additional file 4: **Figure S3**. Functional analysis of HOXD9 in vitro. **(A)** The GC cells (5 × 10^3^) were plated in a tissue culture dish with complete culture medium for 14 days. Cell colonies were visualized after staining with 0.005% crystal violet. ****, *P* < 0.001. **(B)** DNA synthesis in GC cells was measured by EdU incorporation assay at 48 h after the indicated transfection. ****, *P* < 0.001, HOXD9 vs Vector. **(C)** Overexpression of HOXD9 led to a significantly quicker migration at 36 and 72 after transfection. ***, *P* < 0.01 and ****, *P* < 0.001. **(D)** Ectopic expression of HOXD9 led to an increased invasive ability of GC cells. Data are represented as normalized invasion (invasion index) relative to the control cells. ****, *P* < 0.001. The experiments were repeated at least three times. (TIF 470 kb)

Additional file 5: **Figure S4.** HOXD9-RUFY3 axis promotes the growth and invasion of GC cells. **(A)** Soft agar colony formation assays using the indicated cell clones. Quantification of colony numbers is presented. The data are presented as the means ± SD; ****, *P* < 0.001. **(B)** DNA synthesis in GC cells was measured by EdU incorporation assay. ****, *P*< 0.001. (C) The stable HOXD9 transfectants with RUFY3 siRNA1 and siRNA2 led to a significantly slower migration compared with HOXD9-overexpressing cells. ***, *P* **<** 0.01 and ****, *P* < 0.001. **(D)** The stable HOXD9 transfectants with RUFY3 siRNA1 and siRNA2 led to a reduced invasive ability compare with HOXD9-overexpressing cells. ****, *P* < 0.001. (TIF 623 kb)

Additional file 6: **Figure S5.** RUFY3 facilitates HOXD9-mediated cell proliferation and metastasis in GC in vivo. **(A) & (B)** The AGS cells (5 × 10^6^) were injected subcutaneously in the right flanks of nude mice. Images shown were captured on day 25 after injection. **(C) & (D)** Tumor size was measured 5 days after tumor cell inoculation in each group. ****, *P* < 0.001, vector vs. HOXD9 and HOXD9 src-shRNA vs. HOXD9-RUFY3-shRNA, respectively. **(E)** External whole-body fluorescence images of the lung by injection of vector, HOXD9 scr-shRNA and HOXD9-RUFY3-shRNA were obtained 42 days after tail vein injection (*N* = 3). **(F)** White-light images of orthotopic tumors resulting from hepatic metastases of mice obtaining 42 days. Yellow arbitrary polygon indicates primary tumor. Yellow arrows indicate hepatic metastatic lesions. **(G)** The numbers of metastatic lesions in the liver were counted. ***, *P* < 0.01, vector vs. HOXD9; ***, *P* < 0.01, HOXD9 src-shRNA vs. HOXD9-RUFY3-shRNA, respectively. **(H)** Metastatic cancer tissues in the liver were stained with H&E. **(I)-(N)** MMP2 or/and MMP9 expression in tumors derived from AGS cells was determined by qRT-PCR and IHC. ****, *P* < 0.001, vector vs. HOXD9 and HOXD9 src-shRNA vs. HOXD9-RUFY3-shRNA, respectively. Scale bars, 100 μm in L, M & N. (TIF 9599 kb)

## Data Availability

All remaining data are available within the article and supplementary files, or available from the authors upon request.

## Abbreviations

ChIP: chromatin immunoprecipitation
ERK1/2: extracellular signal-regulated protein kinase
GC: gastric cancer
HOX: Homeobox
JNK: c-Jun N-terminal kinase
p38: p38 mitogen-activated protein kinases
qPCR: Real-time quantitative reverse transcription polymerase chain reaction
RLU: relative luciferase unit
RUFY3: RUN and FYVE domain containing 3
Src shRNA: scramble shRNA
TMA: Tissue microarray
