# Supplementary material for: HOXD9 promotes the growth, invasion and metastasis of gastric cancer cells by transcriptional activation of RUFY3
Source: J Exp Clin Cancer Res. 2019 Sep 23;38:412. doi: 10.1186/s13046-019-1399-1 (PMC6755711; doi:10.1186/s13046-019-1399-1)
Supplement: Supplementary file 1 — Supplementary Materials and Methods. Table S1. Primary Primers Used in This Study. (DOCX 23 kb) [file 13046_2019_1399_MOESM1_ESM.docx]

**Supplementary materials**

**Materials and Methods**

Tissue **multi-**array **(**TMA**) and immunohistochemical analysis.**

Briefly, ninety case GC tissues and adjacent non-tumor tissues from patients with GC. The paraffin embedded tissue sections were deparaffinized with xylene and were rehydrated. 5% hydrogen peroxide was used to quench the peroxidase activity. For antigen retrieval the sections were cooked under high pressure by placing the sections in 10 mM sodium citrate buffer (pH 6) in a pressure cooker. Sections were blocked with 4% non-fat dry milk, incubated overnight with primary antibodies at 4°C followed by incubation with the biotin-linked anti-Rabbit IgG (Dako, Copenhagen, Denmark) in combination with the DAB complex. Normal rabbit (Sigma) was used as the isotype controls. The HOXD9 and RUFY3 staining results were classified according to the carcinoma cell staining intensity as follows: 0, negative staining; 1, weak staining; 2, moderate staining; and 3, intense staining. We defined negative- and weak-stained cells as low expressers, and cells that were moderately and intensely stained were considered to be high expressers of this protein. The average score for each sample evaluated by two pathologists (Department of Pathology, Nanfang Hospital, Southern Medical University) was considered as the final IHC score.

**Cell proliferation assay and colony forming assay**

For the proliferation assay, the GC cells were seeded at 5 × 10^3^ cells/well in 96-well plates and incubated at 37°C for 3 days. An aliquot of 10 μl Cell Counting Kit-8 (CCK-8) reagent (Dojindo, Japan) was added to the cells and, following a 3-h incubation, absorbance was measured at 450 nm using a spectrophotometer (Bio-Rad, USA).For the colony forming assay, the four transfectant cells were plated at 100 cells/well in six-well plates, incubated for 2 weeks in RPMI-1640 and stained with crystal violet. Then, images of the stained plates were captured, and colonies containing more than 50 cells were counted. Each treatment was performed in triplicate.

**EdU incorporation assay.**

The GC cells lines were seeded into 6-well dishes at a density of 1 × 10^5^ cells/ml and allowed to adhere overnight. Next, the cells were cultured with 5-ethynyl-2′-deoxyuridine (EdU) for 4 h before detection. The proliferation rate of the cells was then evaluated using a Cell-Light EdU cell proliferation detection kit (RiboBio, Guangzhou, China) according to the manufacturer's instructions.

**ChIP Assay.**

For the ChIP assays, the cancer cells were cultured in three 15 cm dishes and washed in PBS. The genomic DNA and protein were cross-linked by the addition of formaldehyde at a final concentration of 1% directly into the culture medium, followed by incubation for 10 min at room temperature. Then, 0.125 M glycine was added to stop the reaction. The cells were subsequently washed twice with PBS, collected in 1 ml SDS lysis buffer, lysed in 200 ml of SDS lysis buffer supplied with protease inhibitor, and sonicated to generate DNA fragments 200–500 bp long.

Supernatants were precleared using a herring sperm DNA/protein G-Sepharose slurry. Recovered supernatants were incubated with a rabbit anti-HOXD9 antibody or an isotype control immunoglobulin G (IgG) for 2 hours in the presence of herring sperm DNA and protein G-Sepharose beads.Immune complexes were precipitated, washed, and eluted according to the manufacturer’s recommendations (Upstate Biotechnology, Lake Placid, NY).After the DNA-protein cross-linkages were reversed by heating at 65°C for 4 h, the DNA was extracted in a mixture of phenol and chloroform, precipitated with ethanol, and resuspended in 50 ml of 10 mM Tris-HCl and 1 mM EDTA (pH 8.0). An equal volume of each sample was used as a template for PCR amplification of the fragment containing the potential RUFY3 promoter on the immunoprecipitated chromatin.

**Figure Legends**

**Supplementary Figure 1. HOXD9 is overexpressed in tumor tissues.** The expression pattern of HOXD9 mRNA in normal and tumor tissues. HOXD9 mRNA expression in various types of cancer was searched in the firebrowse database (<http://firebrowse.org/>).

**Supplementary Figure 2.** Kaplan-Meier curves for overall survival (OS) from the KM-Plotter database (<http://kmplot.com/analysis/index.php?p=service&start=1>) (Figure 2A) and TCGA dataset (<http://xena.ucsc.edu/public>, Figure 2B).

**Supplementary Figure 3. Functional analysis of HOXD9 in vitro. (A)** The GC cells (5 × 10^3^) were plated in a tissue culture dish with complete culture medium for 14 days. Cell colonies were visualized after staining with 0.005% crystal violet. ****, P < 0.001. **(B)** DNA synthesis in GC cells was measured by EdU incorporation assay at 48 h after the indicated transfection. ****, P < 0.001, HOXD9 vs Vector. **(C)** Overexpression of HOXD9 led to a significantly quicker migration at 36 and 72 after transfection. ***, P < 0.01 and ****, P < 0.001. **(D)** Ectopic expression of HOXD9 led to an increased invasive ability of GC cells. Data are represented as normalized invasion (invasion index) relative to the control cells. ****, P < 0.001. The experiments were repeated at least three times.

**Supplementary Figure 4. HOXD9-RUFY3 axis promotes the growth and invasion of GC cells. (A)** Soft agar colony formation assays using the indicated cell clones. Quantification of colony numbers is presented. The data are presented as the means ± SD; ****, P < 0.001. **(B)** DNA synthesis in GC cells was measured by EdU incorporation assay. ******, P < 0.001.** **(C)** The stable HOXD9 transfectants with RUFY3 siRNA1 and siRNA2 led to a significantly slower migration compared with HOXD9-overexpressing cells. ***, P **<** 0.01 and ****, P < 0.001. **(D)** The stable HOXD9 transfectants with RUFY3 siRNA1 and siRNA2 led to a reduced invasive ability compare with HOXD9-overexpressing cells. ****, P < 0.001.

**Supplementary Figure 5. RUFY3 facilitates HOXD9-mediated cell proliferation and metastasis in GC in vivo. (A) & (B)**The AGS cells (5 × 10^6^) were injected subcutaneously in the right flanks of nude mice. Images shown were captured on day 25 after injection. **(C) & (D)**Tumor size was measured 5 days after tumor cell inoculation in each group. ****, P < 0.001, vector vs. HOXD9 and HOXD9 src shRNA vs. HOXD9-RUFY3-shRNA, respectively. **(E)** External whole-body fluorescence images of the lung by injection of vector, HOXD9-scr-shRNA and HOXD9-RUFY3-shRNA were obtained 42 days after tail vein injection (N = 3). **(F)**

White-light images of orthotopic tumors resulting from hepatic metastases of mice obtaining 42 days. Yellow arbitrary polygon indicates primary tumor. Yellow arrows indicate hepatic metastatic lesions. **(G)**The numbers of metastatic lesions in the liver were counted. ***, P< 0.01, vector vs. HOXD9; ***, P< 0.01, HOXD9 src shRNA vs. HOXD9- RUFY3-shRNA, respectively. **(H)** Metastatic cancer tissues in the liver were stained with H&E. **(I)-(N)** MMP2 or/and MMP9 expression in tumors derived from AGS cells was determined by qRT-PCR and IHC. ****, P< 0.001, vector vs. HOXD9 and HOXD9 src shRNA vs. HOXD9-RUFY3-shRNA, respectively. Scale bars, 100 μm in L, M & N.

**Supplementary Table 1. Primary Primers Used in This Study**

| Experiment | Name | Position or  orientation | Sequence (5’-3’) |
| --- | --- | --- | --- |
| Luciferase  construction | R: | +730 ~ +750 | GAAGATCT GGCGTCAGAGCAGACATGAT (Bgl II) |
|  | L: Rufy3p0 | +351 ~ +371 | GGGGTACC GCCAGATTTTTAAAGCCAGCTA |
|  | L: Rufy3p1-WT | +207 ~ + 226 | GGGGTACC TGAGCAGATCCTGGAAGTGA (Kpn I) |
|  | L: Rufy3p2-WT | -160 ~ -135 | GGGGTACC TCTAACTGCCTCTTTTACCATCTTT - (Kpn I) |
|  | L:  Rufy3p3-WT | -496 ~ -469 | GGGGTACC TTGATAAATACAACAAAATCAAAAAGG (Kpn I) |
| Site-  directed mutagenesis | Rufy3p1-MT | +294 ~ +303 | CGTGTACATTTTTTTTCTGCACAAAGGAGG |
|  | L:  Rufy3p2-MT | -104 ~ -95 | TACGTCTATTCAGTCCTTGATC |
|  | L: Rufy3p3-MT | -345 ~ -336 | AGTGATATATTTAAATTGAAA |
| Chip | Chip 1 | L: +204 ~ +224 | TGAGCAGATCCTGGAAGTGA |
|  |  | R:+385 ~ +404 | TAGGCTTTAAATCCCGCACA |
|  | Chip 2 | L: -193~ -169 | TGAAAGAGGTGAATTATATGCTTG |
|  |  | R: -20 ~ +1 | AACCCACTTCCAGACTGTGC |
|  | Chip 3 | L:-368 ~ -344 | GAAGGAAATTATAAATGCCCAAGA |
|  |  | R:- 193 ~ -169 | CAAGCATATAATTCACCTCTTTCA |
|  | Distant region | L: -4073 ~ -4053 | GTGTGATTCTGGGCCAGTTA |
|  |  | R: -3893 ~ -3873 | GGAGGTTGCAGTAAGCCAAG |

* Underline: HOXD9 binding site mutation
