# Supplementary figures and images for: HOXD9 promotes the growth, invasion and metastasis of gastric cancer cells by transcriptional activation of RUFY3
Source: J Exp Clin Cancer Res. 2019 Sep 23;38:412. doi: 10.1186/s13046-019-1399-1 (PMC6755711; doi:10.1186/s13046-019-1399-1)

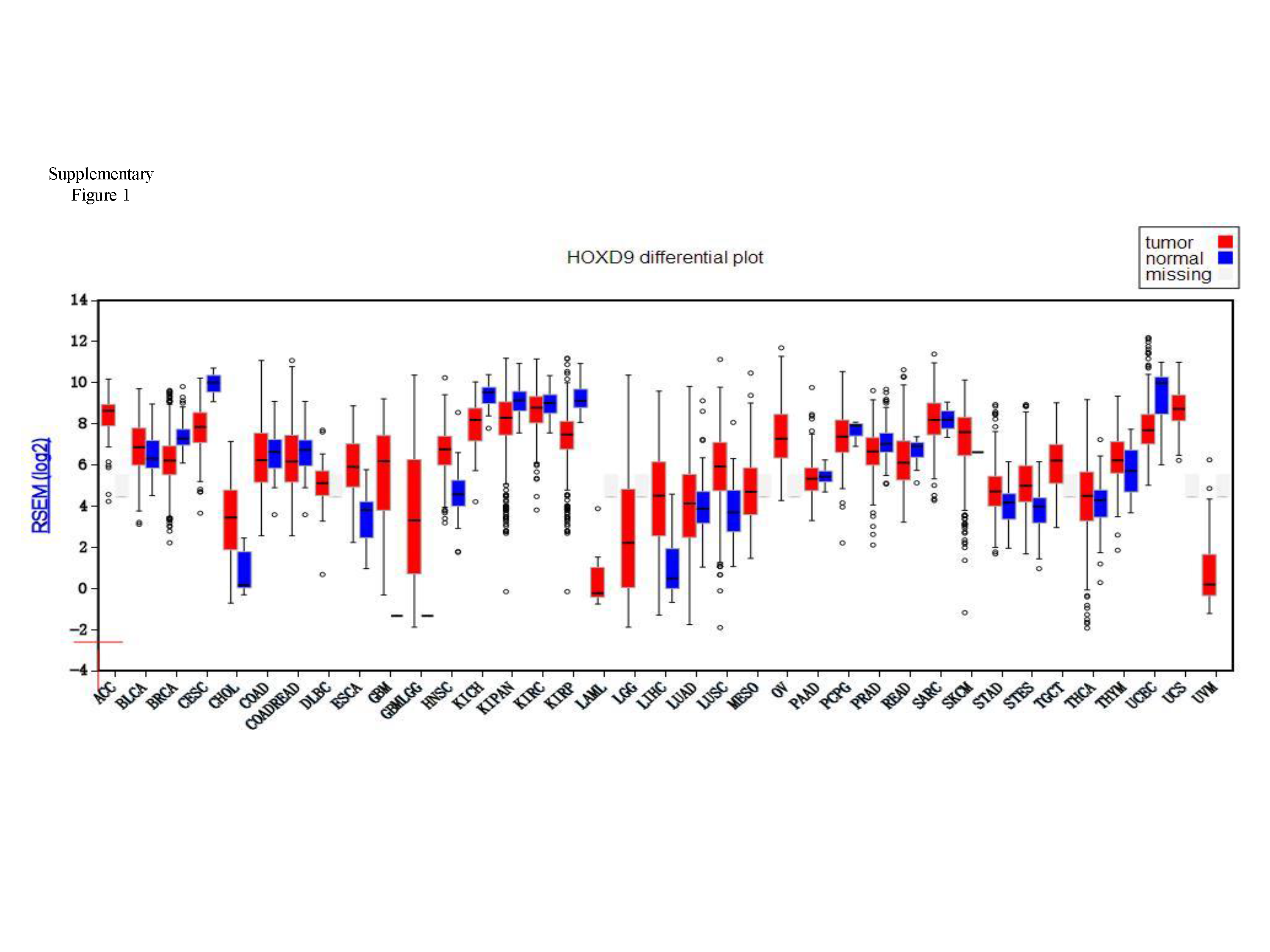

Supplement: Supplementary file 2 — Figure S1. HOXD9 is overexpressed in tumor tissues. The expression pattern of HOXD9 mRNA in normal and tumor tissues. HOXD9 mRNA expression in various types of cancer was searched in the firebrowse database (http://firebrowse.org/). (TIF 3019 kb) [file 13046_2019_1399_MOESM2_ESM.tif]

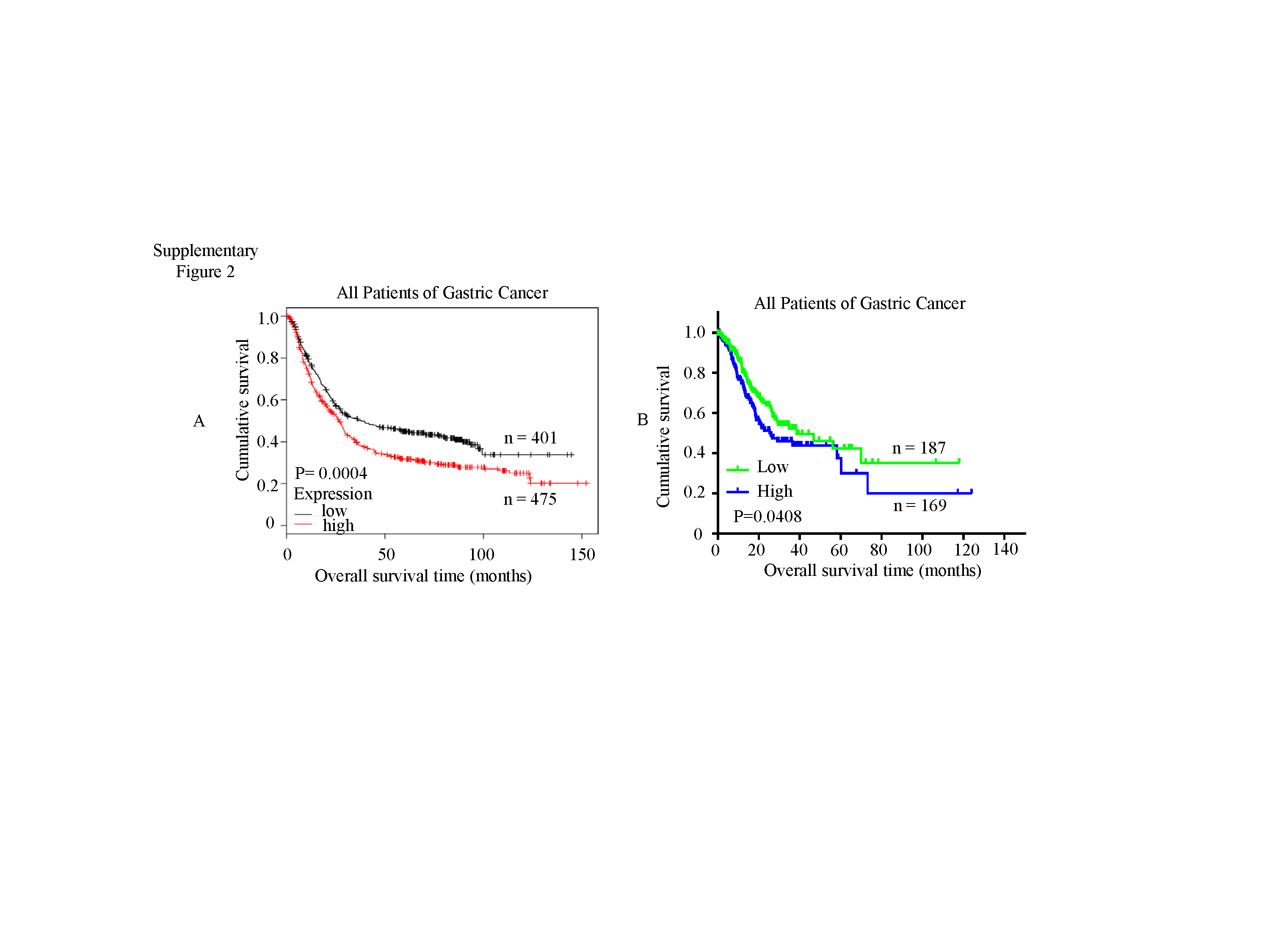

Supplement: Supplementary file 3 — Figure S2. Kaplan-Meier curves for overall survival (OS) from the KM-Plotter database (http://kmplot.com/analysis/index.php?p=service&start=1) (Fig. 2a) and TCGA dataset (http://xena.ucsc.edu/public, Fig. 2b). (TIF 448 kb) [file 13046_2019_1399_MOESM3_ESM.tif]

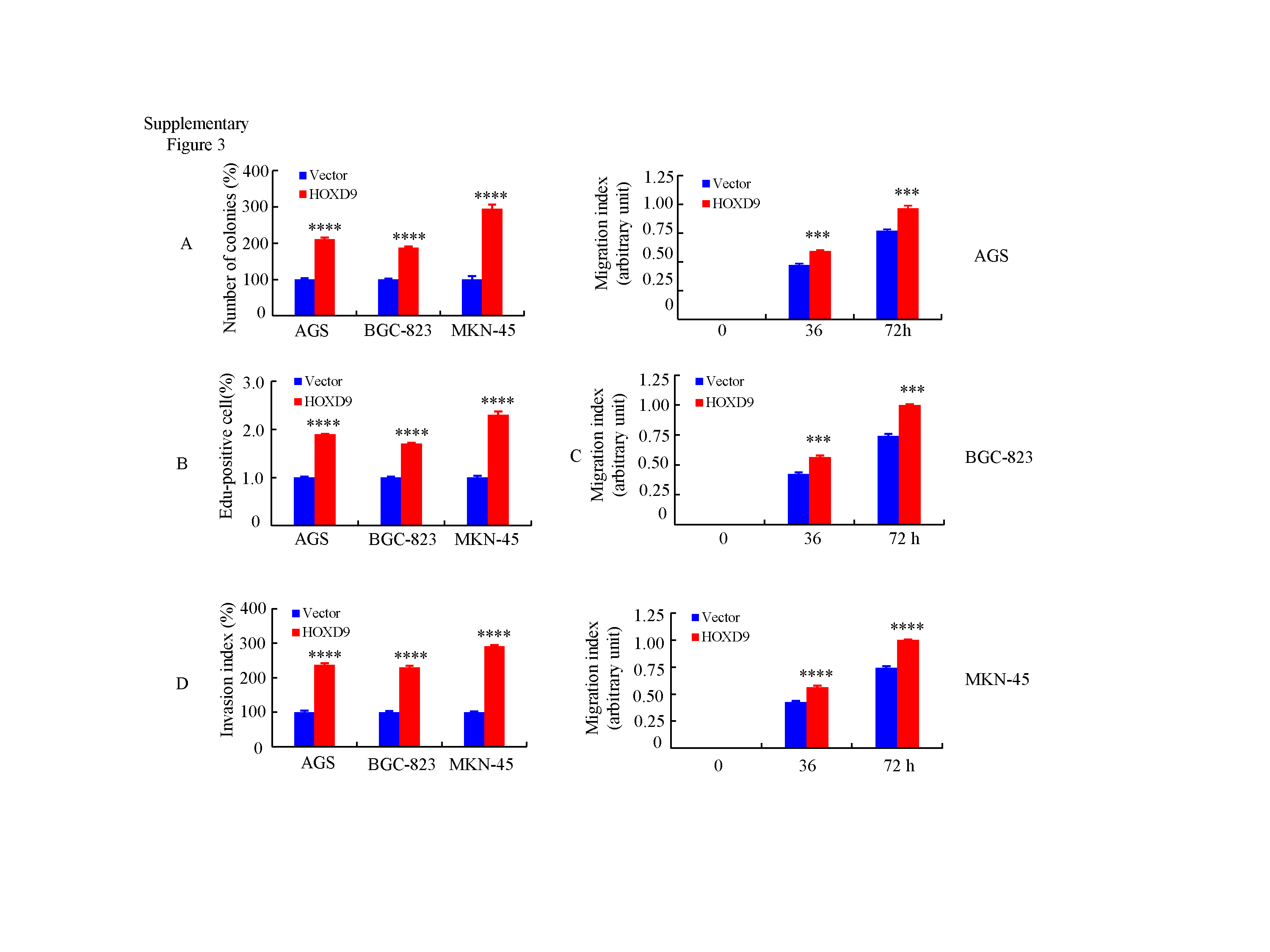

Supplement: Supplementary file 4 — Figure S3. Functional analysis of HOXD9 in vitro. (A) The GC cells (5 × 10^3^) were plated in a tissue culture dish with complete culture medium for 14 days. Cell colonies were visualized after staining with 0.005% crystal violet. ****, P < 0.001. (B) DNA synthesis in GC cells was measured by EdU incorporation assay at 48 h after the indicated transfection. ****, P < 0.001, HOXD9 vs Vector. (C) Overexpression of HOXD9 led to a significantly quicker migration at 36 and 72 after transfection. ***, P < 0.01 and ****, P < 0.001. (D) Ectopic expression of HOXD9 led to an increased invasive ability of GC cells. Data are represented as normalized invasion (invasion index) relative to the control cells. ****, P < 0.001. The experiments were repeated at least three times. (TIF 470 kb) [file 13046_2019_1399_MOESM4_ESM.tif]

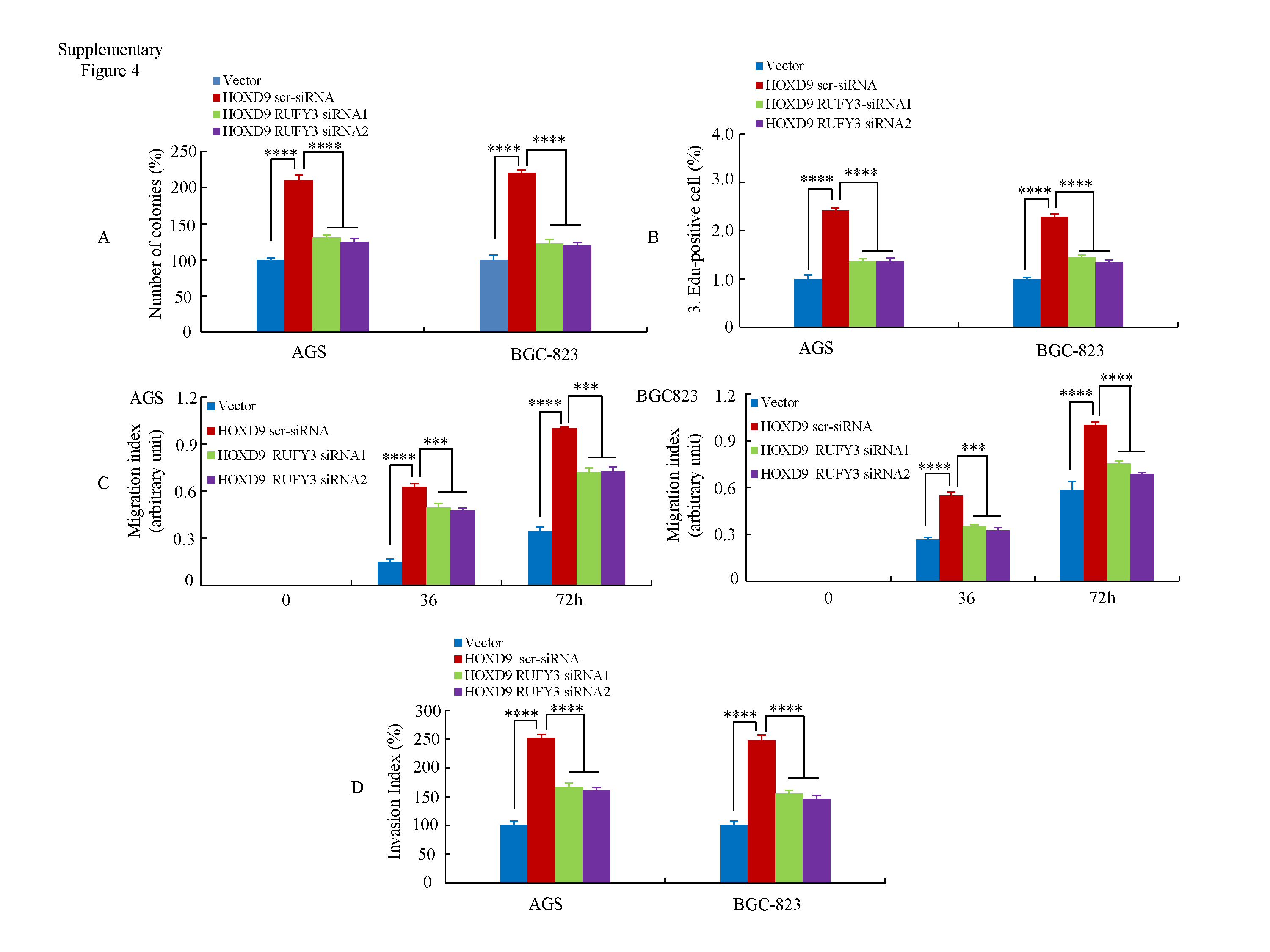

Supplement: Supplementary file 5 — Figure S4. HOXD9-RUFY3 axis promotes the growth and invasion of GC cells. (A) Soft agar colony formation assays using the indicated cell clones. Quantification of colony numbers is presented. The data are presented as the means ± SD; ****, P < 0.001. (B) DNA synthesis in GC cells was measured by EdU incorporation assay. ****, P< 0.001. (C) The stable HOXD9 transfectants with RUFY3 siRNA1 and siRNA2 led to a significantly slower migration compared with HOXD9-overexpressing cells. ***, P < 0.01 and ****, P < 0.001. (D) The stable HOXD9 transfectants with RUFY3 siRNA1 and siRNA2 led to a reduced invasive ability compare with HOXD9-overexpressing cells. ****, P < 0.001. (TIF 623 kb) [file 13046_2019_1399_MOESM5_ESM.tif]

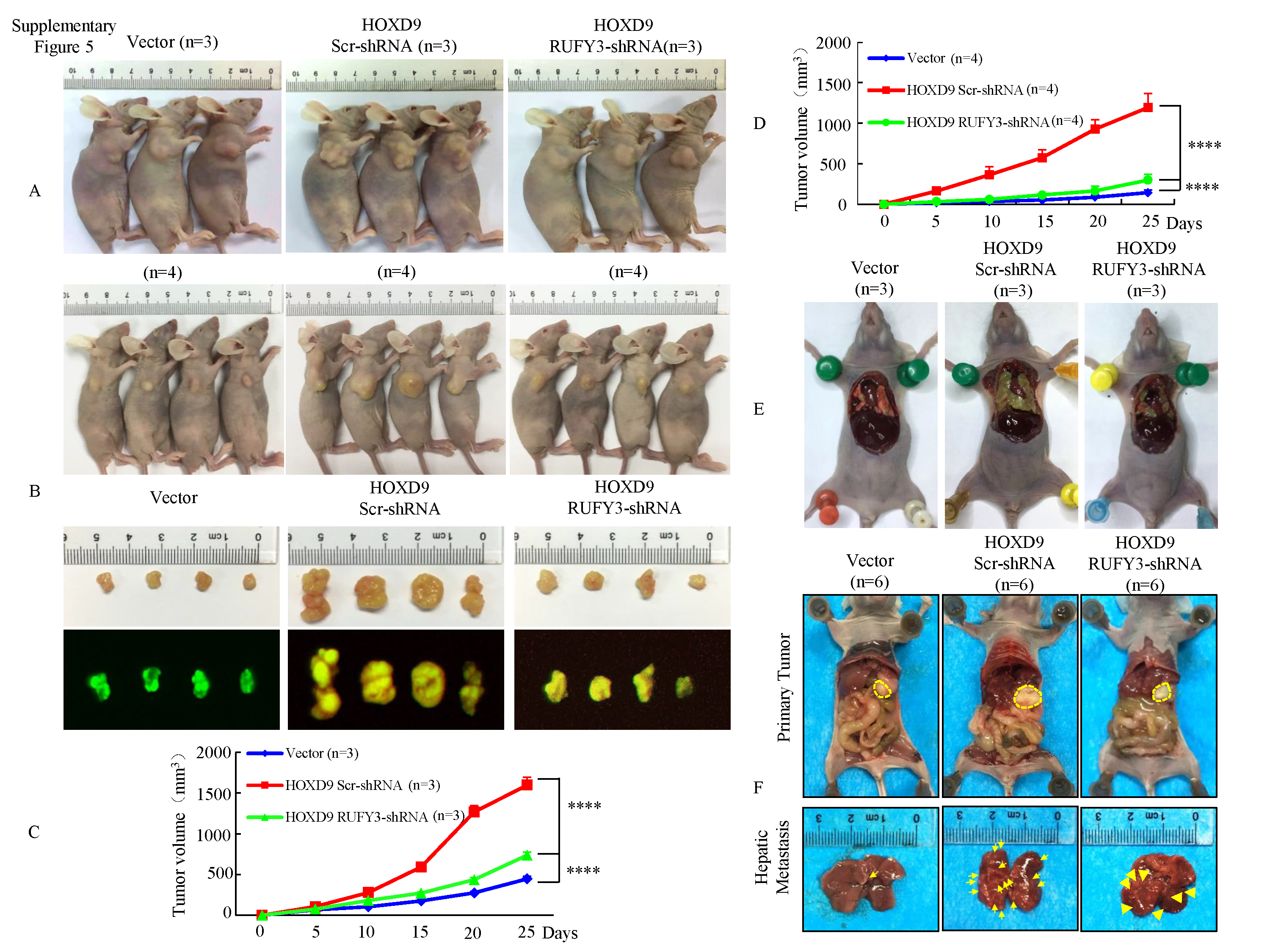

Supplement: Supplementary file 6 — Figure S5. RUFY3 facilitates HOXD9-mediated cell proliferation and metastasis in GC in vivo. (A) & (B) The AGS cells (5 × 10^6^) were injected subcutaneously in the right flanks of nude mice. Images shown were captured on day 25 after injection. (C) & (D) Tumor size was measured 5 days after tumor cell inoculation in each group. ****, P < 0.001, vector vs. HOXD9 and HOXD9 src-shRNA vs. HOXD9-RUFY3-shRNA, respectively. (E) External whole-body fluorescence images of the lung by injection of vector, HOXD9 scr-shRNA and HOXD9-RUFY3-shRNA were obtained 42 days after tail vein injection (N = 3). (F) White-light images of orthotopic tumors resulting from hepatic metastases of mice obtaining 42 days. Yellow arbitrary polygon indicates primary tumor. Yellow arrows indicate hepatic metastatic lesions. (G) The numbers of metastatic lesions in the liver were counted. ***, P < 0.01, vector vs. HOXD9; ***, P < 0.01, HOXD9 src-shRNA vs. HOXD9-RUFY3-shRNA, respectively. (H) Metastatic cancer tissues in the liver were stained with H&E. (I)-(N) MMP2 or/and MMP9 expression in tumors derived from AGS cells was determined by qRT-PCR and IHC. ****, P < 0.001, vector vs. HOXD9 and HOXD9 src-shRNA vs. HOXD9-RUFY3-shRNA, respectively. Scale bars, 100 μm in L, M & N. (TIF 9599 kb) [file 13046_2019_1399_MOESM6_ESM.tif]
